# Oncogenic CLDN1 promotes thyroid cancer progression through TAp63-mediated glycolytic switching

**DOI:** 10.1016/j.isci.2026.117943

**Published:** 2026-11-02

**Authors:** Bowei Zhang, Jinju Wang, Qianxi Zhang, Quanxin Wan

**Affiliations:** 1 Department of thyroid surgery, Sichuan Provincial People’s Hospital, University of Electronic Science and Technology of China, Chengdu, China; 2 School of Medicine, University of Electronic Science and Technology of China, Chengdu, Sichuan 610054, China; 3 Department of Head and Neck Surgery Center, Sichuan Clinical Research Center for Cancer, Sichuan Cancer Hospital and Institute, Sichuan Cancer Center, University of Electromic Science and Technology of China, Chengdu, China

**Keywords:** thyroid cancer, CLDN1, glycolysis, metabolic reprogramming, TAp63 pathway

## Abstract

Thyroid cancer (TC) incidence has risen globally, with advanced cases showing limited treatment options and poor prognosis. Claudin-1 (CLDN1), a tight junction protein, has context-dependent roles in cancer, but its function in TC remains unclear. Integrated bioinformatic analysis of TCGA and GEO datasets identified CLDN1 as a key hub gene upregulated in TC and associated with poor survival. Functional experiments demonstrated that CLDN1 knockdown suppressed TC cell proliferation, migration, and glycolytic metabolism, while its overexpression promoted these effects. Mechanistically, CLDN1 activated the TAp63 pathway, upregulating glycolytic enzymes (HK2, PKM2, and GLUT1) and enhancing glucose uptake, lactate production, and NADPH/NADP^+^ ratio. The glycolysis inhibitor 2-DG reversed CLDN1-driven oncogenic phenotypes *in vitro* and *in vivo*. In xenograft models, CLDN1 overexpression accelerated tumor growth, which was mitigated by 2-DG treatment. Collectively, CLDN1 drives TC progression by reprogramming glycolytic metabolism via the TAp63 pathway, suggesting the CLDN1-glycolysis axis as a potential therapeutic target for TC.

## Highlights

• CLDN1 is upregulated in thyroid cancer and predicts poor patient survival
• CLDN1 promotes thyroid cancer proliferation, migration, and xenograft growth
• CLDN1 reprograms glycolysis via TAp63, elevating HK2, PKM2, and GLUT1
• 2-DG reverses CLDN1-driven glycolysis and tumor growth *in vitro* and *in vivo*

## Graphical abstract

**Figure:**
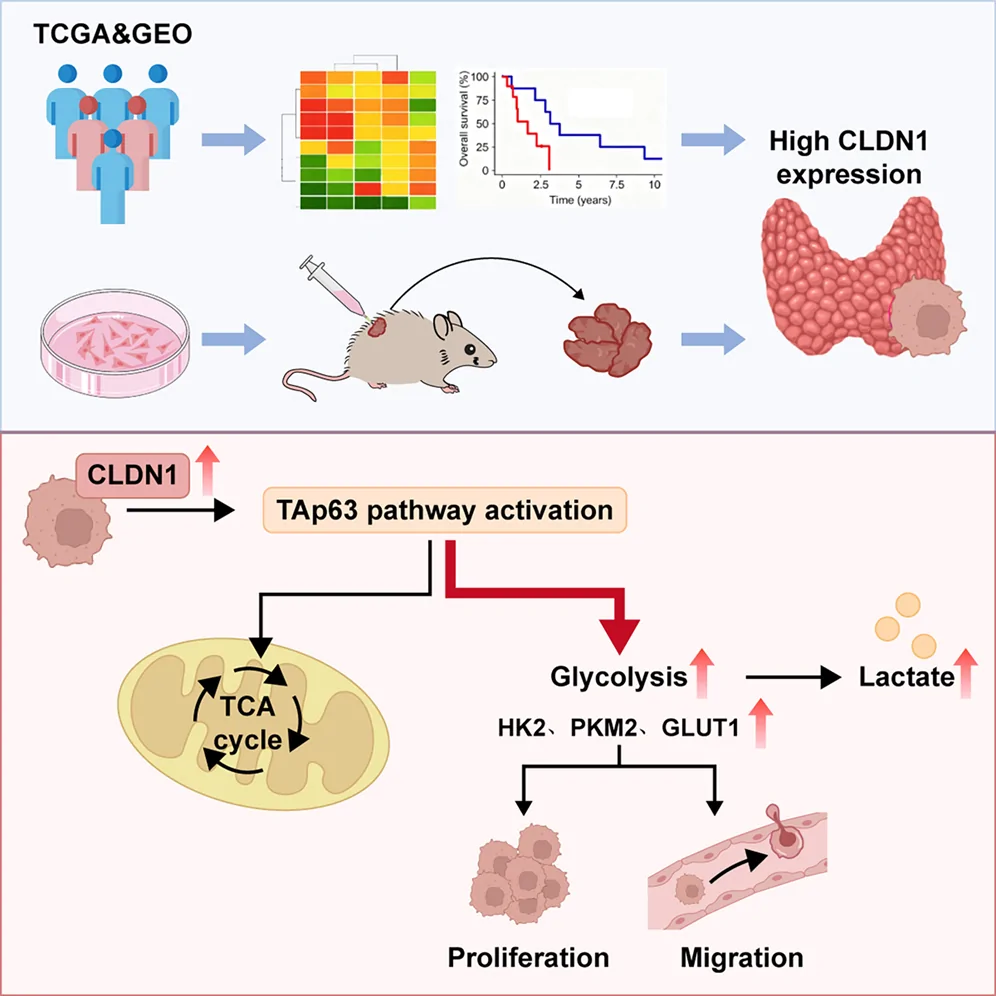

## Teaser

Cancer; Cell biology; Molecular biology

## Introduction

Thyroid cancer (TC) represents the most common malignancy of the endocrine system, with its incidence having increased significantly worldwide over recent decades.^1^ While the majority of TC cases, particularly well-differentiated subtypes, are associated with a favorable prognosis following standard treatment including surgery, radioiodine ablation, and thyroid-stimulating hormone suppression, a subset of patients develops aggressive, recurrent, or metastatic disease.^2^^,^^3^ Treatment options for these patients are limited, often leading to poor outcomes.^4^ This underscores the urgent need to identify novel molecular drivers of TC progression and potential therapeutic targets to improve the management of advanced disease.

Claudin-1 (CLDN1), a key component of tight junctions, plays a critical role in maintaining cell polarity and controlling paracellular permeability.^5^ Beyond its structural function, aberrant expression of CLDN1 has been frequently reported in various cancers; however, its role appears to be context and cancer-type dependent, acting as either a tumor suppressor or an oncogene.^6^^,^^7^^,^^8^ In TC, the expression pattern and functional significance of CLDN1 remain poorly defined and somewhat controversial, warranting further systematic investigation.

A hallmark of cancer, including TC, is metabolic reprogramming, notably the Warburg effect, where cancer cells preferentially utilize glycolysis for energy production even under aerobic conditions.^9^^,^^10^ This metabolic shift not only supports rapid proliferation by providing biomass and energy but also promotes an aggressive phenotype, therapeutic resistance, and immune evasion.^11^^,^^12^^,^^13^ Several key glycolytic enzymes and transporters, such as HK2, PKM2, and GLUT1, have been implicated in TC progression.^14^ However, the upstream regulators that orchestrate this glycolytic switch in TC are not fully elucidated.

In this study, we integrated multi-omics data from public databases and conducted a series of *in vitro* and *in vivo* experiments to investigate the clinical significance, biological functions, and underlying mechanisms of CLDN1 in TC. We aimed to determine whether CLDN1 drives TC malignancy by reprogramming glucose metabolism, thereby positioning the CLDN1-glycolysis axis as a potential therapeutic target for this disease.

## Results

### CLDN1 is a key molecule associated with poor prognosis in TC

To identify key molecules closely related to TC, we integrated gene expression data from TC and normal tissues in the The Cancer Genome Atlas (TCGA) database and Gene Expression Omnibus (GEO) database (GSE29265), performed differential expression analysis, and obtained 443 differentially expressed genes from their intersection (Figures 1A–1C). Using the STRING database, we constructed a protein-protein interaction (PPI) network for these differentially expressed genes. Core module analysis performed in Cytoscape identified 10 candidate genes (Figure 1D). Given its marked overexpression in our analysis, combined with its established role in tumor progression across multiple cancer types, we selected CLDN1 as the primary target for subsequent functional studies.^5^^,^^7^ Compared with normal tissue, CLDN1 expression was significantly upregulated in TC tissue (Figure 1E). To further investigate the relationship between CLDN1 expression and TC prognosis, patients were divided into high-expression and low-expression groups based on CLDN1 expression levels (Figure 1F). Survival analysis was performed by combining follow-up time and survival status. Results revealed significantly shorter overall survival in the high-expression group compared to the low-expression group (Figure 1G, *p* = 0.03), suggesting CLDN1 expression levels may serve as a potential prognostic indicator for TC. In survival prediction analyses at different time points (1, 3, and 5 years), CLDN1 demonstrated superior predictive efficacy within 5 years (Figure 1H). Forest plots demonstrated that CLDN1 expression independently influenced patient survival time (Figure 1I, *p* = 0.027). Validation using microarray samples from 58 TC and adjacent normal tissues confirmed significantly higher CLDN1 expression in cancerous tissues compared to adjacent normal tissues (Figure 1J and Table S1, *p* < 0.05). Moreover, CLDN1 expression levels showed a significant positive correlation with T infiltrate of TC (Tables S2 and S3), indicating that higher expression may correlate with larger primary tumor size and more severe local invasion. Collectively, CLDN1 may play a crucial role in the initiation, progression, and prognosis of TC and holds promise as a potential therapeutic target.

**Figure 1:**
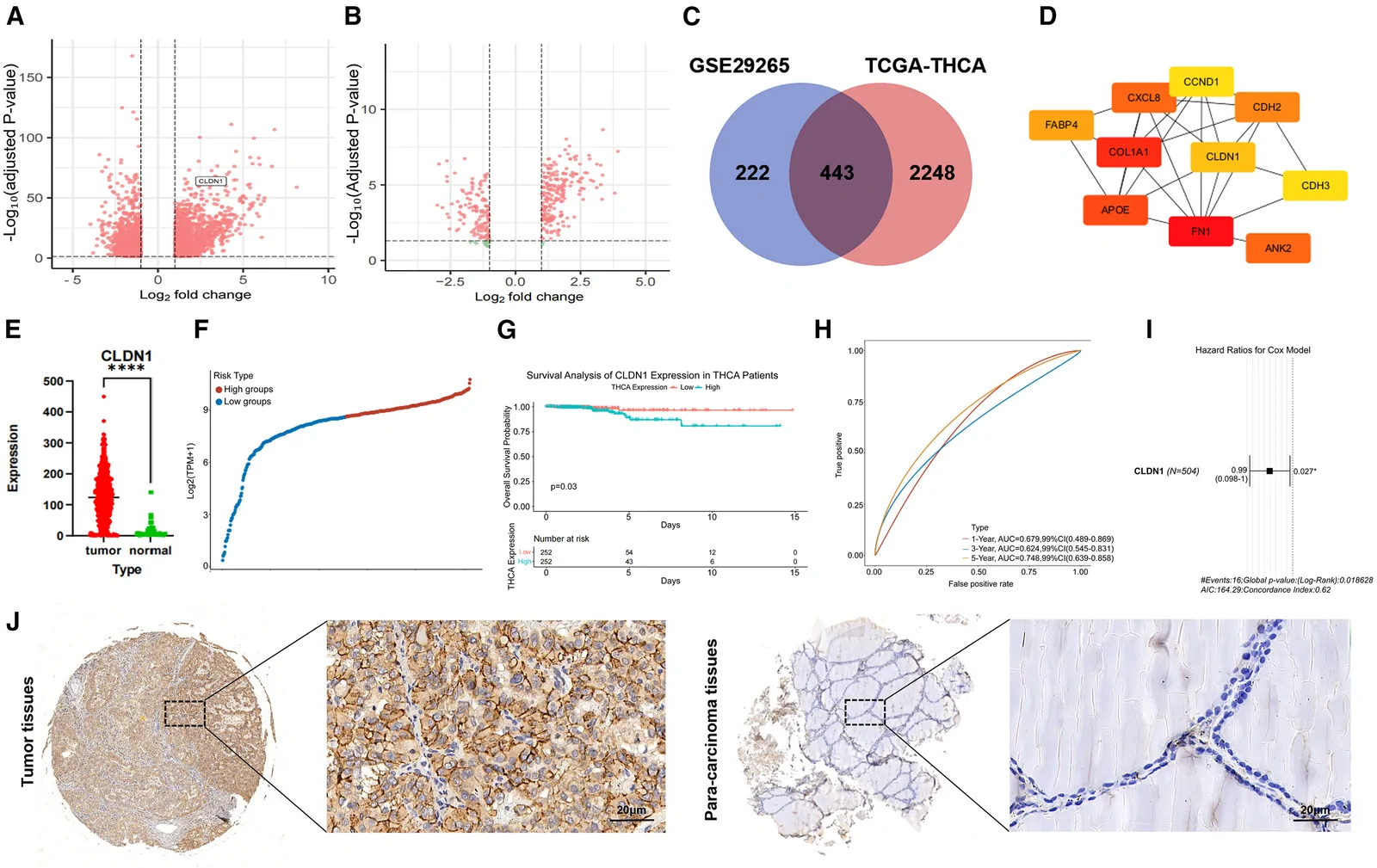
CLDN1 is a key molecule associated with poor prognosis in TC (A) Volcano plot of differential expression genes (DEGs) of TCGA-THCA. (B) Volcano plot of DEGs of GSE29265. (C) Intersection analysis of DEGs between TCGA-THCA and GSE29265. (D) PPI networks of core module genes performed by Cytoscape. (E) Differential expression analysis of CLDN1 between TC and normal tissues. ∗∗∗∗*p* < 0.0001. (F) The distribution of all patients sorted by the expression level of the CLDN1 gene. The red and blue dots represent different expression level groups, with red indicating the high-expression group and blue indicating the low-expression group. (G) Kaplan-Meier analysis demonstrates that high CLDN1 expression correlates with poor overall survival (log-rank test). (H) ROC curves of the predictive ability of the CLDN1 gene for the prognosis of TC patients at different time points (1, 3, and 5 years). (I) Forest plot was used to display the hazard ratio (HR) of the CLDN1 gene in the Cox proportional hazards regression model and its confidence interval. (J) IHC staining of TC and para-carcinoma tissues at 200× magnifications (scale bars, 20 μm).

### CLDN1 promotes proliferation and migration of TC cells

To investigate the role of CLDN1 in TC progression, we first assessed CLDN1 expression levels in the human normal thyroid cell line Nthy-ori 3–1 and multiple TC cell lines. Results showed that both mRNA and protein levels of CLDN1 were significantly higher in papillary thyroid carcinoma (B-CPAP) and follicular thyroid carcinoma (FTC-133) cells than in normal cells (Figures 2A–2C). To further investigate the role of CLDN1, stable CLDN1-knockdown cell lines were generated in B-CPAP and FTC-133 cells, which endogenously express high levels of CLDN1 (Figures 2D and 2E). CCK-8 assays demonstrated that CLDN1 knockdown significantly inhibited TC cell proliferation (Figure 2F). Colony formation assays further demonstrated that CLDN1 knockdown markedly weakened the clonogenic capacity of B-CPAP and FTC-133 cells (Figure 2G). Assessment of the impact of CLDN1 on cell migration via wound healing and Transwell migration assays revealed that CLDN1 deficiency significantly inhibited TC cell migration (Figures 2H and 2I). These results indicate that CLDN1 knockdown significantly inhibits the proliferation and migration of TC cells.

**Figure 2:**
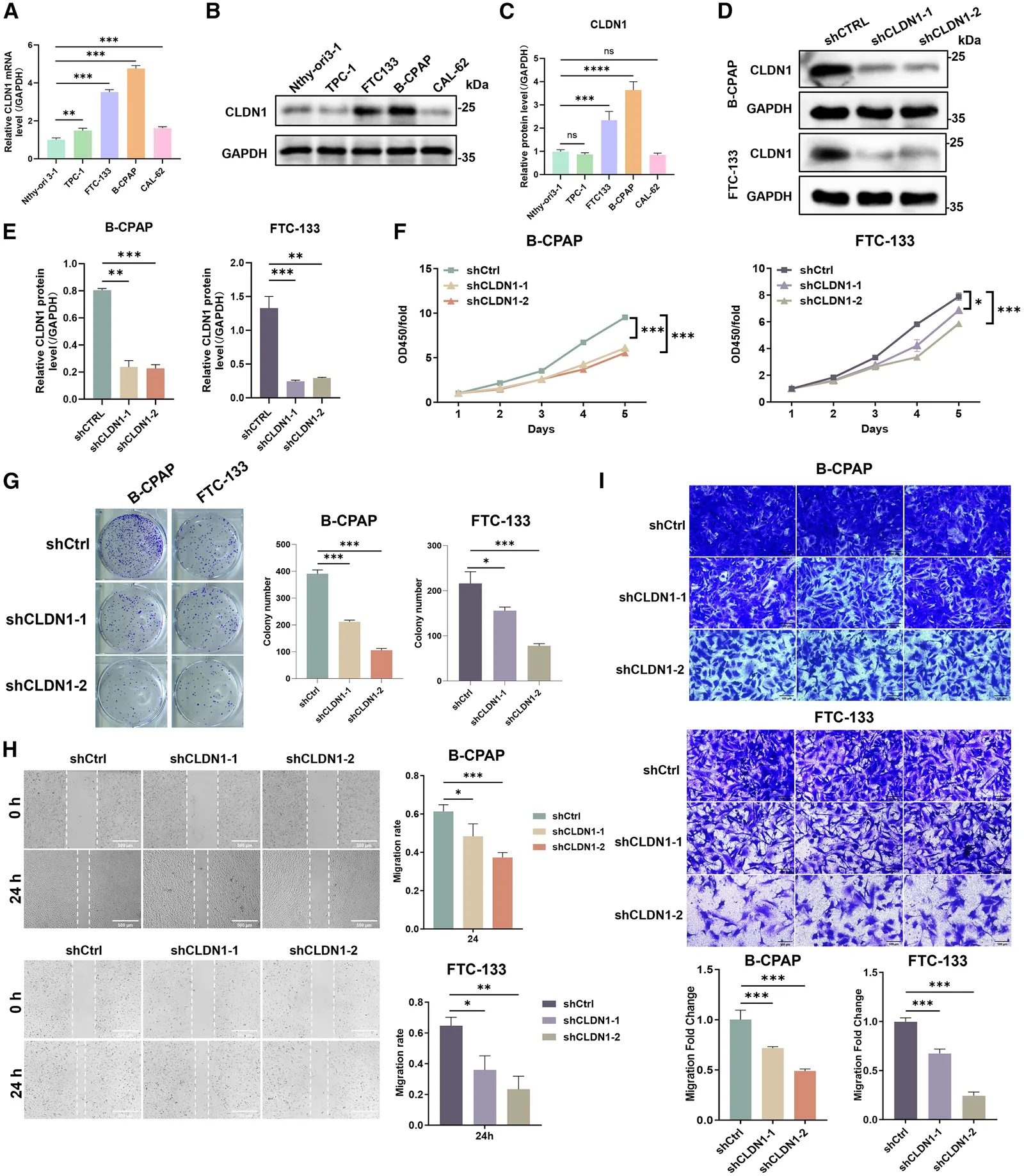
CLDN1 promotes proliferation and migration of TC cells (A) Quantitative analysis of the mRNA expression of CLDN1 in TC cell lines and normal thyroid cell lines. ∗∗*p* < 0.01, ∗∗∗*p* < 0.001. (B and C) Quantitative analysis of the protein expression of CLDN1 in TC cell lines and normal thyroid cell lines. ∗∗∗*p* < 0.001, ∗∗∗∗*p* < 0.0001. ns, no significance. (D and E) Representative western blot images and grayscale analysis of CLDN1 protein expression in B-CPAP and FTC-133 cells transfected with shCtrl or shCLDN1. ∗∗∗*p* < 0.001. (F) Cell proliferation rates in B-CPAP and FTC-133 cells infected with shCtrl and shCLDN1 groups were determined by CCK8 assay. ∗*p* < 0.05, ∗∗∗*p* < 0.001. (G) Representative images of colony formation and the colony number in shCtrl and shCLDN1 groups of B-CPAP and FTC-133 cells. ∗*p* < 0.05, ∗∗∗*p* < 0.001. (H) Representative images of wound healing assay to detect the migration rate in shCtrl and shCLDN1 groups of B-CPAP and FTC-133 cells. (Scale bars, 500 μm). ∗*p* < 0.05, ∗∗*p* < 0.01, ∗∗∗*p* < 0.001. (I) Representative images of Transwell assay and the migratory cells per field in shCtrl and shCLDN1 groups of B-CPAP and FTC-133 cells. (Scale bars, 100 μm). ∗∗∗*p* < 0.001.

### CLDN1 influences glucose metabolism in TC cells via the TAp63 pathway

To further investigate the molecular mechanisms by which CLDN1 regulates TC cell growth, we screened for significantly differentially expressed genes based on TCGA database transcriptome data from TC and normal tissues (Figure 3A) and performed PID pathway enrichment analysis. Results showed that the TAp63 signaling pathway was significantly enriched (Figure 3B). Previous studies have demonstrated that TAp63 promotes glycolytic processes and activates the pentose phosphate pathway (PPP) in tumor cells. For instance, inducing TAp63α expression in p53-deficient osteosarcoma cells enhances glucose uptake and utilization, leading to accumulation of glycolytic intermediates and the end product lactate.^15^^,^^16^ Building on this, we examined the expression of genes within this pathway and found that CLDN1 knockdown significantly downregulated mRNA expression of ITGA3, fatty acid synthase (FAS), ITGB4, PERP, and TAp63 in both B-CPAP and FTC-133 cells (Figure 3C). Similarly, at the protein level, CLDN1 deficiency also led to reduced expression of TAp63, FAS, and ITGA3 (Figure 3D). Further assessment of CLDN1’s impact on key glycolysis proteins revealed that protein expression of HK2, PKM2, and GLUT1 significantly decreased following CLDN1 knockdown (Figure 3E), suggesting inhibition of glycolytic processes. Moreover, CLDN1 knockdown markedly reduced glucose uptake, ATP and lactate production in both B-CPAP and FTC-133 cells, accompanied by an overall decrease in the NADPH/NADP^+^ ratio (Figures 3F–3I). Analysis of cellular metabolic status using a seahorse system revealed reduced extracellular acidification rate (ECAR) and increased oxygen consumption rate (OCR) in CLDN1-deficient cells (Figures 3J and 3K), further confirming impaired glycolysis. These findings demonstrate that CLDN1 deficiency reduces glycolytic activity and PPP metabolic function in TC cells by inhibiting the TAp63 pathway.

**Figure 3:**
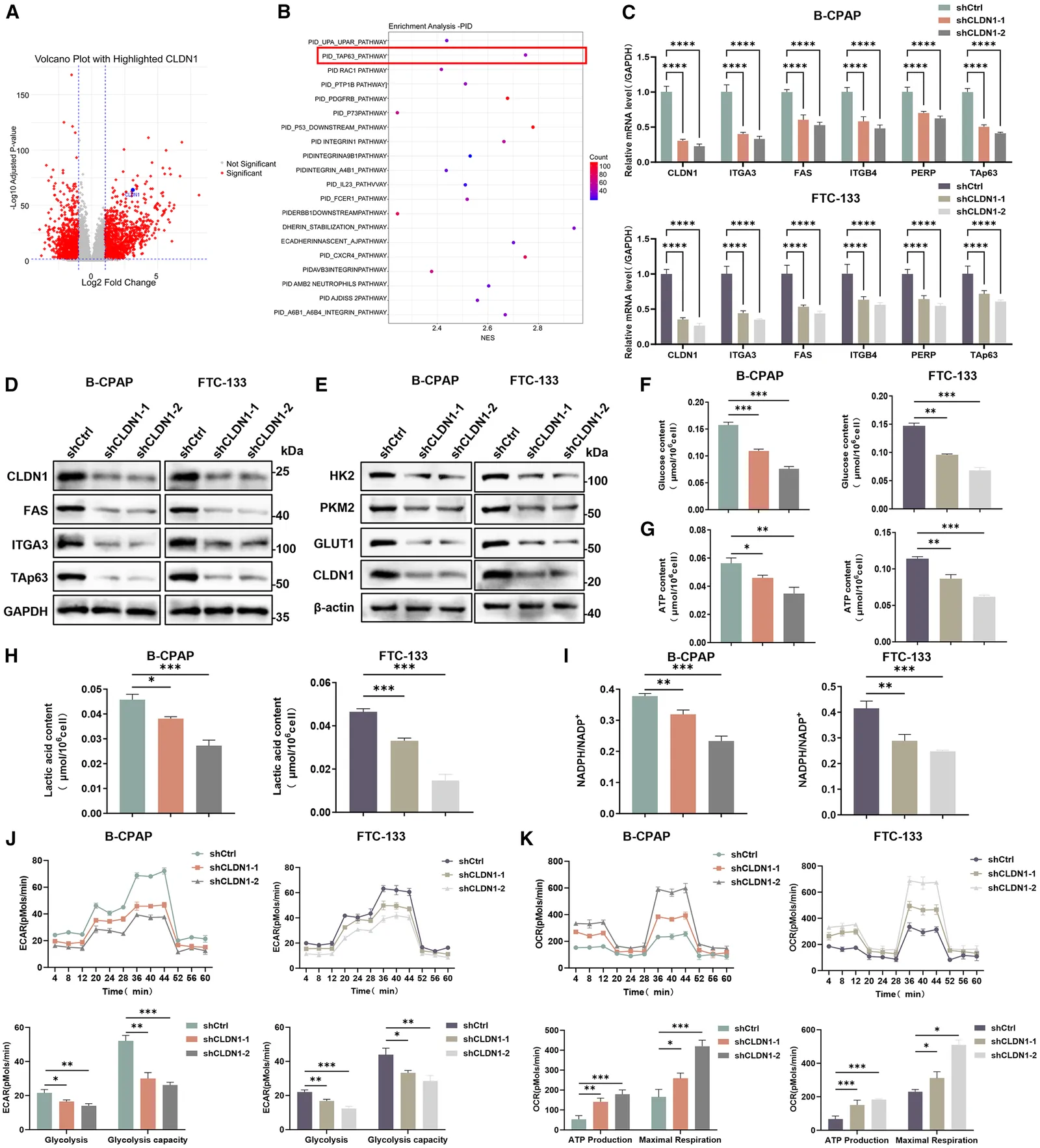
CLDN1 influences glucose metabolism in TC cells via the TAp63 pathway (A) Volcano plot of differential expression genes (DEGs) of TCGA-THCA. (B) Screening of the significantly enriched gene sets of the classical pathway in the PID databases (in order of absolute value of the NES) and plotting of the bubble diagram. (C) qRT-PCR was used to detect the mRNA level of genes in TAp63 pathway in B-CPAP and FTC-133 cells transfected with shCtrl or shCLDN1. ∗*p* < 0.05, ∗∗*p* < 0.01, ∗∗∗*p* < 0.001. (D) Representative western blot analysis of TAp63, CLDN1, FAS, ITGA3 protein in B-CPAP and FTC-133 cells transfected with shCtrl or shCLDN1. (E) Representative western blot analysis of glycolysis related protein in B-CPAP and FTC-133 cells transfected with shCtrl or shCLDN1. (F) Glucose content was detected to show glucose uptake capacity of B-CPAP and FTC-133 cells. ∗∗∗*p* < 0.001. (G) ATP content was detected to show glycolytic activity of B-CPAP and FTC-133 cells. ∗*p* < 0.05. (H) Lactic acid content was detected to show glycolytic activity of B-CPAP and FTC-133 cells. ∗∗∗*p* < 0.001. (I) NADPH/NADP^+^ ratio was tested to show glycolytic activity and PPP metabolic function of B-CPAP and FTC-133 cells. ∗∗*p* < 0.01, ∗∗∗*p* < 0.001. (J) ECAR values were used to show glycolysis capacity of B-CPAP and FTC-133 cells. ∗*p* < 0.05, ∗∗*p* < 0.01, ∗∗∗*p* < 0.001. (K) OCR values were used to show mitochondrial respiratory rate of B-CPAP and FTC-133 cells. ∗*p* < 0.05, ∗∗∗*p* < 0.001.

### CLDN1 promotes TC progression by activating glycolysis

Glycolysis plays a pivotal role in TC progression, not only supplying energy and biosynthetic precursors for rapid cancer cell proliferation but also regulating immune evasion and metastasis.^17^^,^^18^^,^^19^ To investigate whether CLDN1 influences the malignant phenotype of TC by modulating glycolysis, we established an overexpressing CLDN1 model in TC cells and co-treated them with the glycolysis inhibitor 2-Deoxy-D-glucose (2-DG). Results revealed that CLDN1 overexpression significantly enhanced glycolytic activity, manifested as increased expression of glycolytic enzymes, increased glucose uptake and elevated ATP, lactate, and NADPH production (Figures 4A–4E). 2-DG treatment markedly reversed these effects. Further energy metabolism analysis revealed that CLDN1 overexpression caused elevated extracellular ECAR and reduced OCR, changes similarly reversible by 2-DG (Figures 4F and 4G). Functionally, CLDN1 overexpression significantly promoted proliferation and migration in B-CPAP and FTC-133 cells, while 2-DG treatment effectively suppressed CLDN1-induced pro-proliferative and pro-migratory effects (Figures 4H–4J). In summary, our findings demonstrated that CLDN1 promoted TC proliferation and migration by activating the glycolytic pathway, and this effect was reversible by the glycolysis inhibitor 2-DG. This suggested that targeting the CLDN1-glycolysis axis may offer a novel therapeutic strategy for TC.

**Figure 4:**
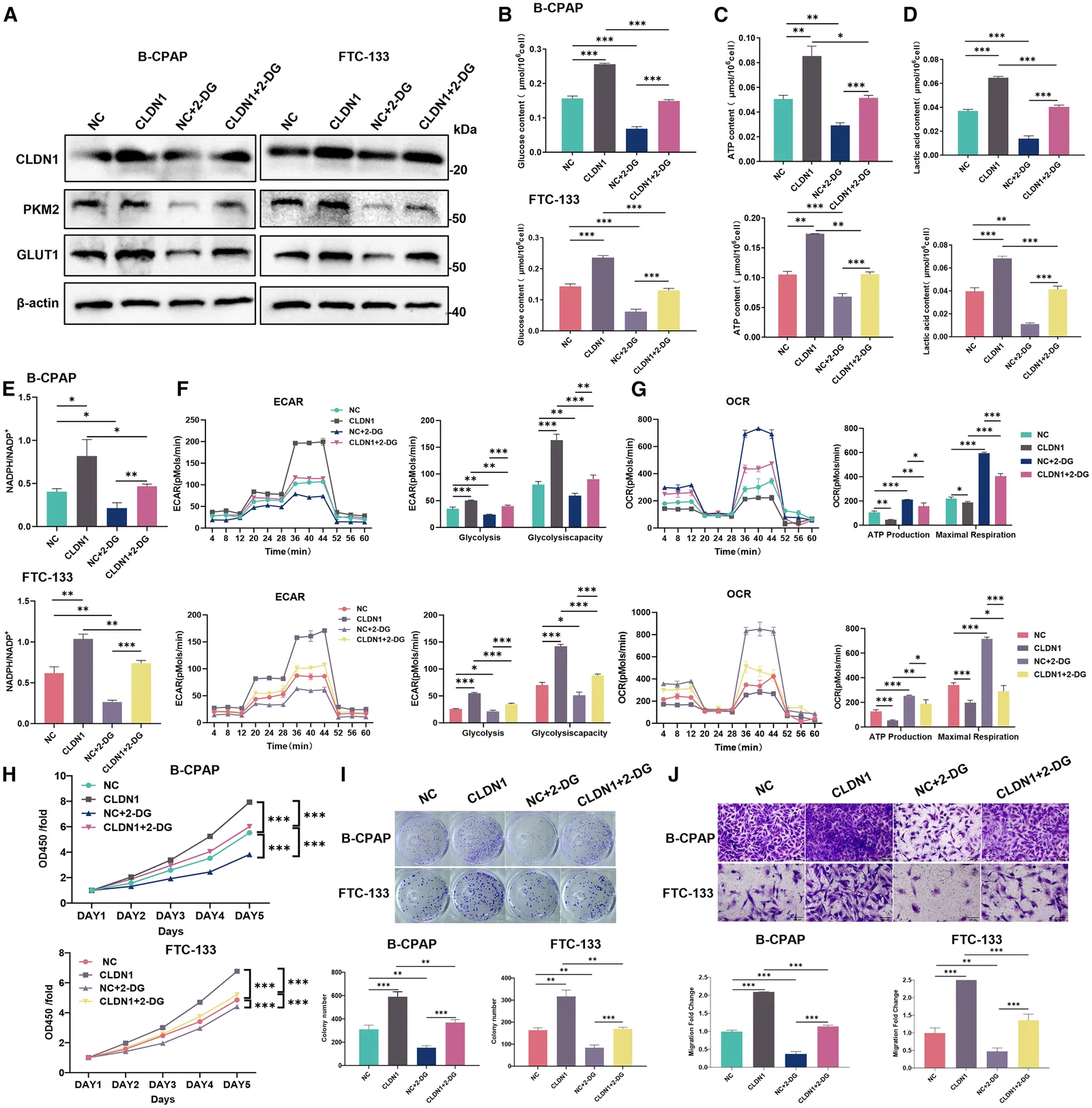
CLDN1 promotes TC progression by activating glycolysis (A) Representative western blot analysis of glycolysis-related protein in B-CPAP and FTC-133 cells treated with CLDN1 overexpression or 2-DG. (B) Glucose content was detected to show glucose uptake capacity of B-CPAP and FTC-133 cells. ∗∗∗*p* < 0.001. (C) ATP content was detected to show glycolytic activity of B-CPAP and FTC-133 cells. ∗*p* < 0.05, ∗∗*p* < 0.01, ∗∗∗*p* < 0.001. (D) Lactic acid content was detected to show glycolytic activity of B-CPAP and FTC-133 cells. ∗∗*p* < 0.01, ∗∗∗*p* < 0.001. (E) NADPH/NADP^+^ ratio was tested to show glycolytic activity and PPP metabolic function of B-CPAP and FTC-133 cells. ∗*p* < 0.05, ∗∗*p* < 0.01, ∗∗∗*p* < 0.001. (F) ECAR values were used to show glycolysis capacity of B-CPAP and FTC-133 cells. ∗*p* < 0.05, ∗∗*p* < 0.01, ∗∗∗*p* < 0.001. (G) OCR values were used to show mitochondrial respiratory rate of B-CPAP and FTC-133 cells. ∗*p* < 0.05, ∗∗*p* < 0.01, ∗∗∗*p* < 0.001. (H) Cell proliferation rates in B-CPAP and FTC-133 cells treated with CLDN1 overexpression or 2-DG were determined by CCK8 assay. ∗∗∗*p* < 0.001. (I) Representative images of colony formation and the colony number in B-CPAP and FTC-133 cells treated with CLDN1 overexpression or 2-DG. ∗∗*p* < 0.01, ∗∗∗*p* < 0.001. (J) Representative images of Transwell assay and the migratory cells per field in B-CPAP and FTC-133 cells treated with CLDN1 overexpression or 2-DG. (Scale bars, 100 μm). ∗∗*p* < 0.01, ∗∗∗*p* < 0.001.

### CLDN1 promotes *in vivo* TC growth via glycolysis

To investigate the role of CLDN1 in TC progression *in vivo*, we established a xenograft tumor model. Results demonstrated that CLDN1 overexpression significantly promoted tumor growth without markedly affecting mouse body weight. Treatment with the glycolysis inhibitor 2-DG significantly suppressed this tumor-promoting effect (Figures 5A–5D), indicating that CLDN1 enhances TC development *in vivo* by activating the glycolysis pathway. Further immunohistochemical (IHC) analysis revealed that Ki67 expression, a proliferation marker, was significantly upregulated in tumors from the CLDN1 overexpression group, an effect that was reversed by 2-DG treatment (Figure 5E). Concurrently, CLDN1 overexpression led to markedly elevated expression levels of glycolysis-related markers, a change similarly suppressed by 2-DG treatment (Figure 5F). In summary, this study integrated clinical data with *in vitro* and *in vivo* evidence to demonstrate that CLDN1 promoted TC proliferation and migration by activating glycolysis. Targeting the CLDN1-glycolysis axis may offer a novel therapeutic strategy for TC.

**Figure 5:**
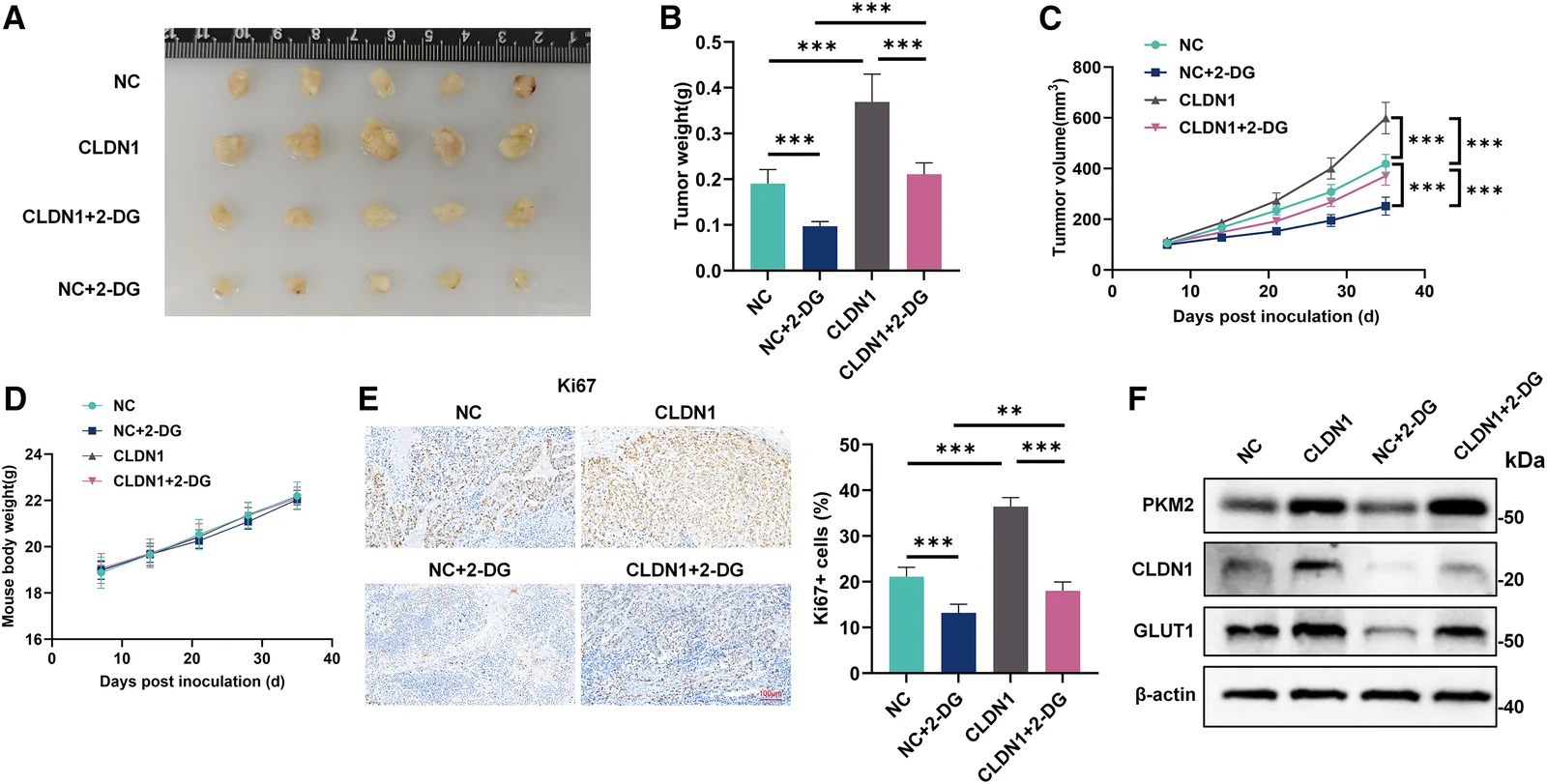
CLDN1 promotes *in vivo* TC growth via glycolysis (A) The photo of tumors removed from mouse models were collected. Tumor weight (B), volume (C), and mouse body weight (D) of xenograft tumors were measured. ∗∗∗*p* < 0.001. (E) Ki-67 staining of tissues treated with CLDN1 overexpression or 2-DG. (Scale bars, 100 μm). ∗∗*p* < 0.01, ∗∗∗*p* < 0.001. (F) Western blot detection of glycolysis related proteins in tissues treated with CLDN1 overexpression or 2-DG.

## Discussion

This study demonstrates that CLDN1 is significantly overexpressed in TC tissues and is associated with poor patient prognosis. Functionally, we found that CLDN1 promotes TC cell proliferation and migration *in vitro* and tumor growth *in vivo*. Mechanistically, we identified that CLDN1 exerts its oncogenic effects primarily by enhancing glycolytic metabolism through the TAp63 signaling pathway. Inhibition of glycolysis with 2-DG effectively abrogated the tumor-promoting effects of CLDN1, both in cellular models and in a xenograft setting. These findings collectively suggest that CLDN1 is a critical regulator of TC progression and that targeting the CLDN1-glycolysis axis may represent a novel therapeutic strategy.

Metabolic reprogramming is widely recognized as a critical factor in the development and progression of TC, making glycolysis a promising therapeutic target.^20^^,^^21^ Studies have shown that glycolysis-related genes can serve as potential biomarkers for TC, and prognostic models based on glycolysis risk scores have been successfully developed to accurately predict recurrence in patients with TC.^10^^,^^22^ Furthermore, inhibiting glycolysis through various signaling pathways, such as mitogen-activated protein kinase (MAPK)/CREB and hypoxia-inducible factor 1 alpha (HIF-1α)/YAP, has been demonstrated to suppress TC growth and metastasis.^23^^,^^24^ Consistently, our results indicate that CLDN1 knockdown downregulated key glycolytic enzymes (HK2, PKM2, and GLUT1), reduced metabolic flux (including glucose uptake, lactate production, and ECAR), and impaired the proliferative and migratory capacities of cancer cells. Notably, the regulatory link between CLDN1 and glycolysis has also been observed in other cancers. For example, in gastric cancer, Xu et al. employed machine learning to reveal significant heterogeneity in glycolytic activity, with more aggressive cells exhibiting enhanced glycolysis, and CLDN1 was defined as one of nine hub genes with strong diagnostic potential.^25^ These findings position CLDN1 as a novel upstream regulator of glycolytic metabolism in TC, offering a mechanistic basis for targeting the CLDN1-glycolysis axis.

In this study, we identified the TAp63 pathway as a downstream effector of CLDN1 in promoting TC progression. TAp63, a transcriptionally active isoform encoded by the TP63 gene and a core member of the p53 family, is classically recognized as a tumor suppressor.^26^ Extensive studies have established that downregulation or loss of function of TAp63 is closely associated with cancer progression and poor prognosis in various malignancies, including colon cancer, breast cancer, head, and neck squamous cell carcinoma.^27^^,^^28^^,^^29^ Its tumor-suppressive mechanisms include upregulating miR-133b to inhibit RhoA signaling and directly repressing the transcription of the oncogene MYCN.^27^ Furthermore, mutant p53 can promote tumorigenesis by inhibiting TAp63 function.^30^ These studies have revealed the significant role of TAp63 in the tumor suppression network.

However, the biological effects of TAp63 are not invariant; its functions appear to be precisely regulated by cell type, tumor microenvironment, and upstream signaling pathways. For example, recent research has confirmed that HIPK4 can inhibit the transcriptional activity of TAp63 by phosphorylating its Ser395 residue, thereby attenuating its tumor-suppressive function.^31^ This suggests that the ultimate functional output of TAp63 may depend on its specific modification states and interaction networks. In this study, we found that TAp63 exhibits an atypical function associated with metabolic reprogramming. In TC, TAp63 is highly expressed, and its expression level positively correlates with the activation of the glycolytic metabolic pathway and tumor progression. This suggests that signaling driven by CLDN1 may convert TAp63 from a tumor suppressor into a driver of metabolic reprogramming. This potential conversion could occur through alterations in its transcriptional co-regulators or the induction of specific post-translational modifications, thereby activating a transcriptional program that promotes glycolysis. Consistent with our findings, cancer cells overexpressing TAp63 demonstrate enhanced glycolytic activity.^32^ Nevertheless, this mechanism does not contradict the classical tumor-suppressive role of TAp63. Instead, it highlights its functional plasticity in acquiring pro-tumorigenic capabilities under specific oncogenic signaling contexts, thereby offering a novel perspective for understanding the complex and multifaceted roles of the p63 family in cancer.

In conclusion, we have identified CLDN1 as a key oncoprotein that drives TC progression by activating glycolysis via the TAp63 pathway. Our work not only elucidates a novel mechanistic link between cell adhesion and metabolism but also provides a strong rationale for developing therapies targeting glycolysis in CLDN1-high TCs. Future research should focus on elucidating the detailed molecular mechanisms upstream and downstream of CLDN1 and exploring the efficacy of specific glycolysis inhibitors in preclinical models of advanced TC.

### Limitations of the study

Despite these significant findings, our study has several limitations. Firstly, the precise molecular mechanism by which the membrane protein CLDN1 regulates glycolysis, particularly how it initiates the intracellular signaling cascade leading to TAp63 pathway activation, remains to be fully elucidated. Secondly, our *in vivo* validation was conducted in immunodeficient mouse models. This precludes the analysis of both metastasis and the potential role of CLDN1 in modulating the tumor immune microenvironment through its metabolic effects. Given the importance of lactate and other glycolytic intermediates in immune suppression, this represents an important avenue for future research.^33^^,^^34^ Thirdly, while we focused on the TAp63 axis, CLDN1 may regulate other oncogenic signaling pathways contributing to TC progression. A more comprehensive analysis, for instance using proteomic or phospho-proteomic approaches, is needed to reveal such additional mechanisms and could address the current lack of proteomic data for CLDN1. Finally, the clinical correlation data, while supportive, are derived from retrospective analyses. Prospective studies with larger cohorts are needed to firmly establish CLDN1 as a robust prognostic biomarker in TC. Future studies should aim to dissect the upstream signaling mechanism by which CLDN1 regulates cellular metabolism. This could involve identifying direct binding partners or intermediary kinases that relay the signal from CLDN1 at the membrane to transcriptional regulators such as TAp63 in the nucleus. Furthermore, the functional consequence of this axis specifically on tumor metastasis requires dedicated analysis to complement the current findings on tumor growth.

## Resource availability

### Lead contact

Requests for further information and resources should be directed to and will be fulfilled by the lead contact, Quanxin Wan (15881005411@163.com).

### Materials availability

This study did not generate new unique reagents.

### Data and code availability

• The gene expression profiles and corresponding clinical data for TC analyzed in this study were obtained from TCGA via the GDC data portal (https://portal.gdc.cancer.gov/) and the NCBI GEO database under the accession code GEO: GSE29265.
• This paper did not report original code.
• Any additional information will be made available upon reasonable request to the lead contact.

## Acknowledgments

This study was supported by the Research Fund of Sichuan Provincial People’s Hospital (no. 2023QN10).

## Author contributions

Q.W., B.Z., and J.W. performed investigation, data curation, and formal analysis; J.W. and Q.Z. validated data; Q.W. supervised the study and managed fundings; B.Z. and Q.Z. wrote the draft of manuscript. All authors reviewed and approved the final manuscript.

## Declaration of interests

The authors declare no competing interests.

## STAR★Methods

### Key resources table

**Table:** 

REAGENT or RESOURCE	SOURCE	IDENTIFIER
**Antibodies**		

Claudin-1 (CLDN1)	Abcam	Cat# ab211737; RRID: AB_3082989
Hexokinase II (HK2)	Cell Signaling Technology	Cat# 2867; RRID: AB_2232946
Pyruvate kinase M2 (PKM2)	Cell Signaling Technology	Cat# 4053; RRID: AB_1904096
Glucose transporter 1 (GLUT1)	Abcam	Cat# ab115730; RRID: AB_10903230
Fatty acid synthase (FAS)	Cell Signaling Technology	Cat# 3180; RRID: AB_2100796
Integrin alpha-3 (ITGA3)	Santa Cruz Biotechnology	Cat# sc-7312; RRID: AB_627819
TAp63 (TP63, TA isoform) antibody	Abcam	Cat# ab124762; RRID: AB_10971840
Ki-67 rabbit mAb [D2H10]	Cell Signaling Technology	Cat# 9027; RRID: AB_2636984
β-Actin mouse mAb [AC-15]	Sigma-Aldrich	Cat# A5441; RRID: AB_476744
HRP-conjugated goat anti-rabbit IgG	Proteintech	#SA00001-2; RRID: AB_2722564
HRP-conjugated goat anti-mouse IgG	Proteintech	#SA00001-1; RRID: AB_2722565

**Biological samples**		

Commercial tissue microarray containing human TC tissues and paired adjacent normal tissues (*n* = 58)	Shanghai Outdo Biotech (HThyP120CS03)	N/A

**Deposited data**		

TCGA-THCA RNA-seq and clinical data	NCI GDC	https://portal.gdc.cancer.gov/
TC microarray dataset	NCBI GEO	GEO: GSE29265
Analyzed data generated in this study	This paper	Available from the lead contact

**Chemicals, peptides, and recombinant proteins**		

RPMI-1640	Gibco	Cat# 11875093
DMEM (high glucose)	Gibco	Cat# 11965092
FBS	Gibco	Cat# 10099141
Penicillin-streptomycin	Gibco	Cat# 15140122
TRIzol reagent	Invitrogen	Cat# 15596026
RIPA lysis buffer	Beyotime	Cat# P0013B
Protease	Roche	Cat# 04693116001
phosphatase inhibitor cocktail	Roche	Cat# PhosSTOP 04906837001
PVDF membrane	Merck Millipore	Cat# IPVH00010
ECL substrate	Merck Millipore	Cat# WBKLS0500
2-Deoxy-D-glucose (2-DG)	Sigma-Aldrich	Cat# D8375; CAS: 154–17–6
Puromycin	Sigma-Aldrich	Cat# P8833
Polybrene	Sigma-Aldrich	Cat# H9268
Sodium pentobarbital	Sigma-Aldrich	Cat# P3761
4% Paraformaldehyde	Beyotime	Cat# P0099
Crystal violet	Sigma-Aldrich	Cat# C0775
Seahorse XF Glycolysis Stress Test Kit	Agilent	Cat# 103020–100
Cell Mito Stress Test Kit	Agilent	Cat# 103015–100

**Critical commercial assays**		

CCK-8	Dojindo	Cat# CK04
BCA Protein Assay Kit	Pierce (Thermo Fisher)	Cat# 23225
PrimeScript RT Reagent Kit with gDNA Eraser	Takara	Cat# RR047A
TB Green Premix Ex Taq II	Takara	Cat# RR820A
Glucose Uptake	Abcam	Cat# ab136955
L-Lactate	Abcam	Cat# ab65331
NADP-NADPH Assay Kits (Colorimetric)	Abcam	Cat# ab65349
ATP Assay Kit	Beyotime	Cat# S0026
DAB substrate kit	ZSGB-BIO	Cat# ZLI-9018

**Experimental models: Cell lines**		

Nthy-ori 3–1	Chinese Academy of Sciences Cell Bank	RRID: CVCL_2659
TPC-1	Chinese Academy of Sciences Cell Bank	RRID: CVCL_6298
FTC-133	Chinese Academy of Sciences Cell Bank	RRID: CVCL_1219
B-CPAP	Chinese Academy of Sciences Cell Bank	RRID: CVCL_0153
CAL-62	Chinese Academy of Sciences Cell Bank	RRID: CVCL_1112
HEK293T	ATCC	Cat# CRL-3216; RRID: CVCL_0063

**Experimental models: organisms/strains**		

BALB/c nude mice, female, 4 weeks old	Vital River Laboratory Animal Technology (Beijing)	IACUC No. 2025801

**Oligonucleotides**		

shCLDN1-1	Genomeditech	5′-TTCTTGCAGGTCTGGCTATTT-3′
shCLDN1-2	Genomeditech	5′-GTCAATGCCAGGTACGAATTT-3′
shNC	Genomeditech	5′-TTCTCCGAACGTGTCACGT-3′
Primers for qPCR CLDN1 F	Sangon Biotech	5′-CCAGTCAATGCCAGGTACGA-3′
Primers for qPCR CLDN1 R	Sangon Biotech	5′-GCTGGAAGGTGCAGGTTTTG-3′
Primers for qPCR TAp63 F	Sangon Biotech	5′-CAGCAGTTGTGTTGGAGGGA-3′
Primers for qPCR TAp63 R	Sangon Biotech	5′-CCAGGACTTGCCCATCTCTG-3′
Primers for qPCR ITGA3 F	Sangon Biotech	5′-GGCCTGCCAAGCTAATGAGA-3′
Primers for qPCR ITGA3 R	Sangon Biotech	5′-GTAGCCGGTGATTTACCATGC-3′
Primers for qPCR FAS F	Sangon Biotech	5′-GTCCAAGACACAGCAGAACAG-3′
Primers for qPCR FAS R	Sangon Biotech	5′-CTGCAAGAGTACAAAGATTGGCT-3′
Primers for qPCR ITGB4 F	Sangon Biotech	5′-GGTGGAGGAGAACCTGAACG-3′
Primers for qPCR ITGB4 R	Sangon Biotech	5′-TTGCTTTTTCCCGGCATTGG-3′
Primers for qPCR PERP F	Sangon Biotech	5′-CTACGAGGAGGGCTGTCAGA-3′
Primers for qPCR PERP R	Sangon Biotech	5′-ATGATCTGGAACACAGCAGCC-3′
Primers for qPCR GAPDH F	Sangon Biotech	5′-GTGGAAGGACTCATGACCACAG-3′
Primers for qPCR GAPDH R	Sangon Biotech	5′-CAGCTCAGGGATGACCTTGC-3′

**Recombinant DNA**		

PGMLV-SC5	Genomeditech	GM-LC-143598
PGMLV-SC5-shCLDN1	This paper	N/A
pLVX-puro	Takara (Clontech)	Cat# 632164
pLVX-puro-CLDN1	This paper	N/A
psPAX2	Addgene	RRID: Addgene_12260
pMD2.G	Addgene	RRID: Addgene_12259

**Software and algorithms**		

GraphPad Prism 9.0	GraphPad	RRID: SCR_002798
R 4.2.1	R Project	RRID: SCR_001905
STRING v11.5	STRING	https://string-db.org
Cytoscape 3.9.1 (MCODE)	Cytoscape	RRID: SCR_003032
ImageJ	NIH	RRID: SCR_003070
Wave Desktop	Agilent	N/A
Image Lab	Bio-Rad	N/A

**Other**		

Seahorse XF96 Analyzer	Agilent	RRID: SCR_026641
ChemiDoc MP	Bio-Rad	17001402; RRID: SCR_019037
QuantStudio 5	Applied Biosystems	A28570; RRID: SCR_020240
Transwell (8 μm)	Corning	3422

### Experimental model and study participant details

#### Human participants

Not applicable.

#### Mice

All animal experiments were conducted in strict accordance with the ARRIVE guidelines 2.0 and approved by the Institutional Animal Care and Use Committee (IACUC) of Sichuan Provincial People’s Hospital (Approval Number: 2025801) and conducted in accordance with the NIH guidelines. Four-week-old female BALB/c nude mice were purchased from Vital River Laboratory Animal Technology Co., Ltd. (Beijing, China).

#### Cell lines

Cell Line: Nthy-ori 3–1; Species/Origin: human, immortalized normal thyroid follicular epithelial cell line; Maintenance and Care: Cells were cultured in RPMI-1640 medium (Gibco) supplemented with 10% (v/v) fetal bovine serum (FBS, Gibco) and 1% penicillin-streptomycin.

Cell Line: TPC-1; Species/Origin: human, papillary thyroid carcinoma cell line; Maintenance and Care: Cells were cultured in RPMI-1640 or DMEM medium (Gibco) supplemented with 10% (v/v) fetal bovine serum (FBS, Gibco) and 1% penicillin-streptomycin.

Cell Line: FTC-133; Species/Origin: human, follicular thyroid carcinoma cell line; Maintenance and Care: Cells were cultured in DMEM medium (Gibco) supplemented with 10% (v/v) fetal bovine serum (FBS, Gibco) and 1% penicillin-streptomycin.

Cell Line: B-CPAP; Species/Origin: human, papillary thyroid carcinoma cell line; Maintenance and Care: Cells were cultured in RPMI-1640 or DMEM medium (Gibco) supplemented with 10% (v/v) fetal bovine serum (FBS, Gibco) and 1% penicillin-streptomycin.

Cell Line: CAL-62; Species/Origin: human, anaplastic thyroid carcinoma cell line; Maintenance and Care: Cells were cultured in DMEM medium (Gibco) supplemented with 10% (v/v) fetal bovine serum (FBS, Gibco) and 1% penicillin-streptomycin.

All cell lines were purchased from the American Type Culture Collection (ATCC) or the Chinese Academy of Sciences Cell Bank (Shanghai, China). All cells were cultured at 37°C in a humidified atmosphere of 5% CO_2_. All cell lines were authenticated by short tandem repeat (STR) profiling and confirmed to be negative for mycoplasma contamination.

### Method details

#### Bioinformatic analysis and data acquisition

Gene expression profiles and corresponding clinical data for TC were downloaded from The Cancer Genome Atlas (TCGA, https://portal.gdc.cancer.gov/) and the Gene Expression Omnibus (GEO) database (GSE29265). Differential expression analysis between TC tissues and normal tissues was performed using the “DESeq2” package in R (for TCGA RNA-seq data) or the “limma” package in R (for GEO microarray data). Genes with an adjusted *p*-value (padj) < 0.05 and |log2(Fold Change)| > 1 were considered differentially expressed genes (DEGs). The common DEGs from both datasets were selected for further analysis.

A protein-protein interaction (PPI) network for the common DEGs was constructed using the STRING database (https://string-db.org/, version 11.5) with a confidence score >0.4. The network was then imported into Cytoscape software (version 3.9.1) for visualization and module analysis. The Molecular Complex Detection (MCODE) plugin was used to identify the most significant module (core module) and hub genes. Survival analysis based on CLDN1 expression was conducted using the “survival” and “survminer” R packages. Patients were stratified into high and low expression groups based on the median expression value of CLDN1.

#### Cell transfection

For gene knockdown, short hairpin RNAs (shRNAs) targeting CLDN1 and a non-targeting scrambled control (shNC) were designed and cloned into a lentiviral vector (PGMLV-SC5). For gene overexpression, the full-length human CLDN1 cDNA was cloned into a lentiviral expression vector (pLVX-puro). Lentiviruses were packaged in 293T cells using the psPAX2 and pMD2.G plasmids. Target cells were infected with the lentiviruses in the presence of 8 μg/mL polybrene. Stable cell lines were selected using 2 μg/mL puromycin for at least one week. Knockdown and overexpression efficiencies were confirmed by quantitative real-time PCR (qRT-PCR) and western blotting.

#### RNA extraction and quantitative real-time PCR (qRT-PCR)

Total RNA was extracted from cells using TRIzol reagent (Invitrogen) according to the manufacturer’s instructions. cDNA was synthesized using the PrimeScript RT reagent Kit with gDNA Eraser (Takara). qRT-PCR was performed on a QuantStudio 5 Real-Time PCR System (Applied Biosystems) using TB Green Premix Ex Taq II (Takara). GAPDH was used as an internal control. The relative gene expression was calculated using the 2ˆ(-ΔΔCt) method.

#### Western blotting

Cells were lysed in RIPA buffer (Beyotime) supplemented with protease and phosphatase inhibitors (Roche). Protein concentration was determined using a BCA Protein Assay Kit (Pierce). Equal amounts of protein were separated by SDS-PAGE and transferred onto PVDF membranes (Millipore). The membranes were blocked with 5% non-fat milk and then incubated overnight at 4°C with primary antibodies against CLDN1 (1:1000, Abcam, ab211737), HK2 (1:1000, CST, #2867), PKM2 (1:1000, CST, #4053), GLUT1 (1:1000, Abcam, ab115730), FAS (1:1000, CST, #3180), ITGA3 (1:1000, Santa Cruz, sc-7312), GAPDH (1: 10000, Proteintech, 60004-1-Ig) and β-actin (1:5000, Sigma, A5441). After incubation with appropriate HRP-conjugated secondary antibodies, protein bands were visualized using an Enhanced Chemiluminescence (ECL) kit (Millipore) and imaged with a ChemiDoc MP Imaging System (Bio-Rad).

#### Cell proliferation and colony formation assay

Cell proliferation was assessed using the Cell Counting Kit-8 (CCK-8, Dojindo) according to the manufacturer’s instructions. Briefly, 2000 cells per well were seeded in a 96-well plate. At the indicated time points (0, 24, 48, 72, 96 h), 10 μL of CCK-8 reagent was added to each well and incubated for 2 h. The absorbance was measured at 450 nm using a microplate reader.

For the colony formation assay, 1000 cells were seeded into each well of a 6-well plate and cultured for 10–14 days. The colonies were fixed with 4% paraformaldehyde and stained with 0.1% crystal violet. Colonies containing more than 50 cells were counted manually.

#### Wound healing and transwell migration assay

For the wound healing assay, cells were seeded in 6-well plates and grown to confluence. A sterile 200 μL pipette tip was used to create a scratch. The cells were then washed with PBS and cultured in serum-free medium. Images of the wound were taken at 0 h and 24 h under a microscope. The migration distance was measured using ImageJ software.

Cell migration was also evaluated using Transwell chambers (8 μm pore size, Corning). Briefly, 5 × 10^4^ cells in 200 μL of serum-free medium were seeded into the upper chamber. The lower chamber was filled with 600 μL of medium containing 20% FBS as a chemoattractant. After 24 h of incubation, the cells that migrated to the lower surface of the membrane were fixed, stained with crystal violet, and counted under a microscope.

#### Glycolysis detection

Glucose uptake was measured using a Glucose Uptake Assay Kit (Colorimetric, Abcam, ab136955) following the manufacturer’s protocol. Intracellular ATP levels were determined using an ATP Assay Kit (Beyotime, S0026). Lactate production in the cell culture supernatant was measured using a Lactate Assay Kit (Colorimetric, Abcam, ab65331). The NADPH/NADP^+^ ratio was assessed using an NADP/NADPH Assay Kit (Colorimetric, Abcam, ab65349).

#### Seahorse XF96 extracellular flux analyzer

The extracellular acidification rate (ECAR) and oxygen consumption rate (OCR) were measured using a Seahorse XF96 Analyzer (Agilent Technologies). For the Glycolysis Stress Test, cells were seeded in XF96 plates and incubated in a CO2-free incubator for 1 h prior to the assay. The assay was performed by sequentially injecting glucose, oligomycin, and 2-Deoxy-D-glucose (2-DG) according to the standard protocol. For the Mito Stress Test, cells were sequentially treated with oligomycin, FCCP, and rotenone/antimycin A. Data were analyzed using the Wave Desktop software.

#### *In vivo* xenograft tumor model

B-CPAP cells stably overexpressing CLDN1 or vector control were subcutaneously injected into the flanks of the mice (5 × 10^6^ cells per mouse). One week after inoculation, mice in the treatment group received intraperitoneal injections of 2-DG (500 mg/kg, dissolved in PBS) every other day, while the control group received PBS. Tumor volume was measured every 3 days and calculated using the formula: Volume = (length × widthˆ2)/2. After 4 weeks, the mice were euthanized by intraperitoneal injection of an overdose of sodium pentobarbital (150 mg/kg), and the tumors were harvested, weighed, and prepared for immunohistochemical analysis.

#### Immunohistochemistry (IHC)

Tumor tissues were fixed in 4% paraformaldehyde, embedded in paraffin, and sectioned at 4 μm thickness. After deparaffinization and antigen retrieval, the sections were incubated with primary antibodies against Ki67 (1:200, CST, #9027) and CLDN1 (1:200, Abcam, ab211737) overnight at 4°C. Subsequently, the sections were incubated with a biotinylated secondary antibody and then with HRP-streptavidin. The signal was developed using a DAB substrate kit (ZSG KD IO), and the sections were counterstained with hematoxylin. Images were captured using a microscope, and the staining intensity was quantified using ImageJ software.

### Quantification and statistical analysis

All statistical analyses were performed using GraphPad Prism software (version 9.0) or R software (version 4.2.1). Data are presented as mean ± standard deviation (SD) from at least three independent experiments. Differences between two groups were analyzed using the unpaired two-tailed Student’s *t* test. Comparisons among multiple groups were performed using one-way analysis of variance (ANOVA) followed by Tukey’s post hoc test. The correlation between CLDN1 expression and clinicopathological features was analyzed using the Chi-square test. Survival curves were plotted using the Kaplan-Meier method and compared by the log rank test. A *p*-value <0.05 was considered statistically significant.

## Supplemental information

Document S1. Tables S1–S3

Document S2. Uncropped and unedited raw WB images.pdf
