# Supplementary material for: Oncogenic CLDN1 promotes thyroid cancer progression through TAp63-mediated glycolytic switching
Source: iScience. 2026 Nov 2;29(11):117943. doi: 10.1016/j.isci.2026.117943 (PMC13755985; doi:10.1016/j.isci.2026.117943)
Supplement: Supplementary file 1 [file mmc1.pdf]

iScience, Volume *29*

Supplemental information

Oncogenic CLDN1 promotes thyroid cancer progression through TAp63-mediated glycolytic switching

Bowei Zhang, Jinju Wang, Qianxi Zhang, and Quanxin Wan

Supplementary information

Table S1. Expression patterns in thyroid cancer tissues and para-carcinoma tissues revealed in immunohistochemistry analysis, related to Figure 1

A expression	Tumor tissue		Para-carcinoma tissue		p value
	Cases	Percentage	Cases	Percentage	
Low	33	57.9%	47	81.0%	P<0.05
High	24	42.1%	11	19.0%	

Table S2. Relationship between CLDN1 expression and tumor characteristics in patients with thyroid cancer, related to Figure 1

Features	No. of patients	CLDN1 expression		p value
		low	high	
All patients	57	33	24	
Age (years)				0.4
< 63	52	31	21	
≥ 63	5	2	3	
Gender				0.476
Male	23	12	11	
Female	34	21	13	
Stage				0.377
1	48	29	19	

2	9	4	5	
T Infiltrate				0.041
T1	28	20	8	
T2	24	11	13	
T3	3	2	1	
T4	2	0	2	
lymphatic metastasis (N)				0.962
N0	29	17	12	
N1	27	16	11	
Metastasis				1
M0	57	33	24	
M1	0	0	0	

Table S3. Spearman correlation analysis of CLDN1 expression and tumor characteristics in patients with thyroid cancer, related to Figure 1

		CLDN1
T Infiltrate	Spearman correlation	0.273
	Significance (two-tailed)	0.04
	N	57
