# Supplementary material for: Oncogenic CLDN1 promotes thyroid cancer progression through TAp63-mediated glycolytic switching
Source: iScience. 2026 Nov 2;29(11):117943. doi: 10.1016/j.isci.2026.117943 (PMC13755985; doi:10.1016/j.isci.2026.117943)
Supplement: Supplementary file 2 [file mmc2.pdf]

Fig.2B

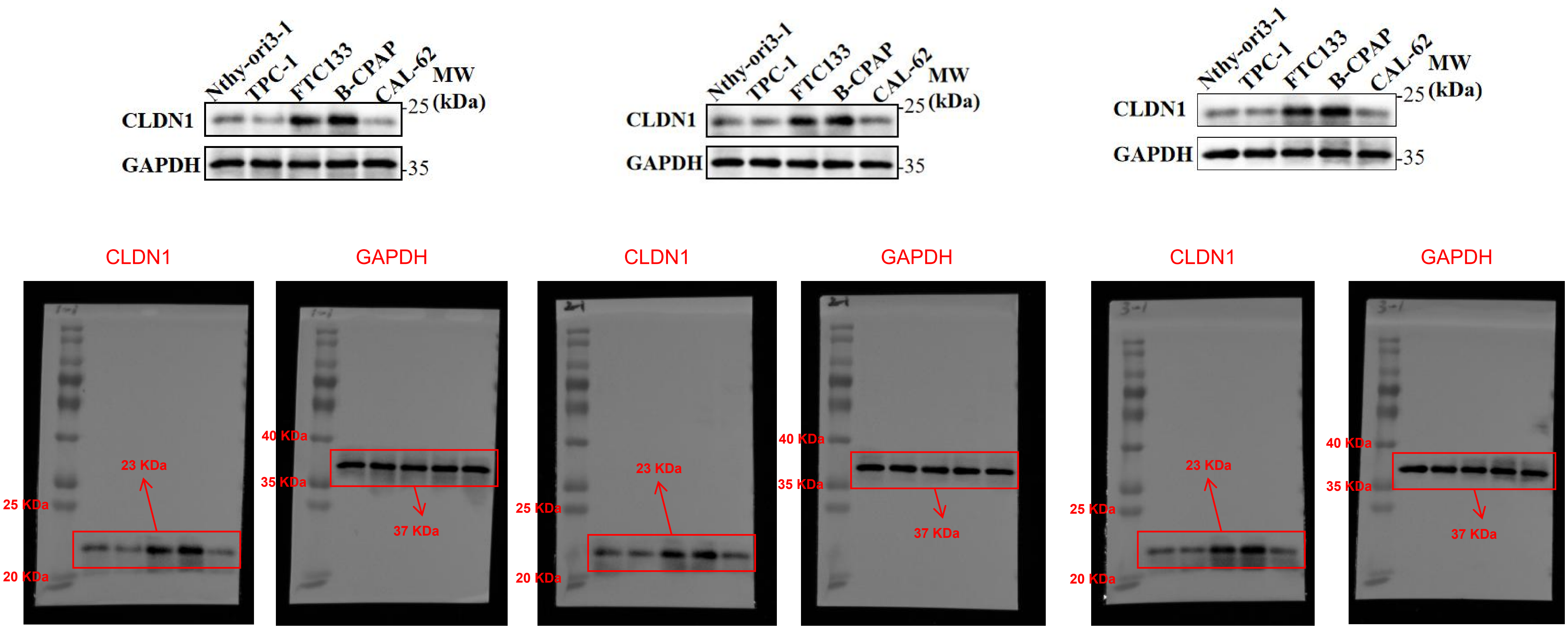

Nthy-ori3-1
TPC-1
FTC133
B-CPAP
CAL-62
MW (kDa)
CLDN1
GAPDH
25
35
Nthy-ori3-1
TPC-1
FTC133
B-CPAP
CAL-62
MW (kDa)
CLDN1
GAPDH
25
35
Nthy-ori3-1
TPC-1
FTC133
B-CPAP
CAL-62
MW (kDa)
CLDN1
GAPDH
25
35
CLDN1
GAPDH
CLDN1
GAPDH
CLDN1
GAPDH
23 KDa
25 KDa
20 KDa
40 KDa
35 KDa
37 KDa
23 KDa
25 KDa
20 KDa
40 KDa
35 KDa
37 KDa
23 KDa
25 KDa
20 KDa
40 KDa
35 KDa
37 KDa

Fig.2D

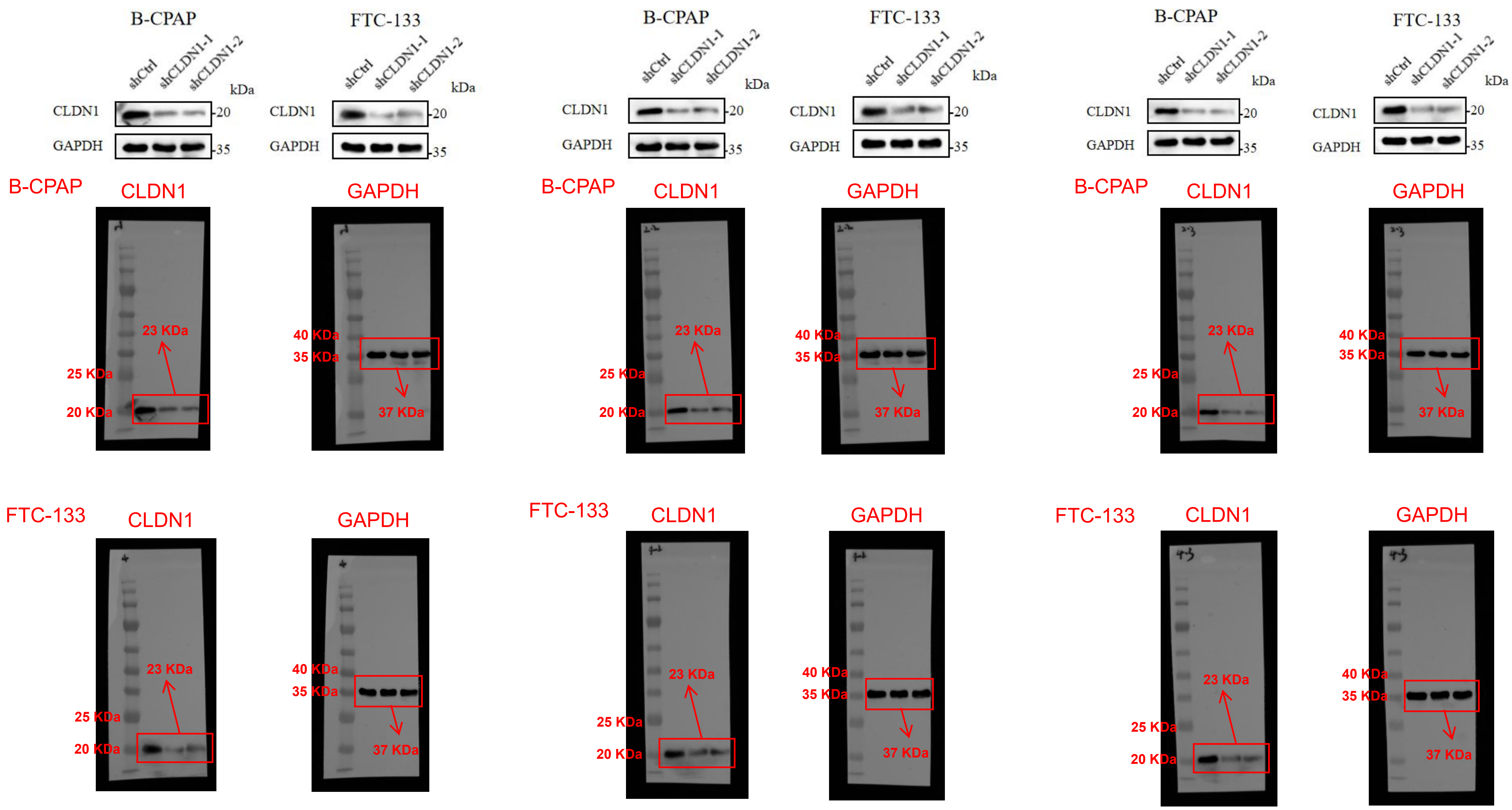
B-CPAP
FTC-133
shCtrl
shCLDN1-1
shCLDN1-2
kDa
CLDN1
GAPDH
20
35
B-CPAP
CLDN1
GAPDH
23 KDa
25 KDa
20 KDa
40 KDa
35 KDa
37 KDa
FTC-133
CLDN1
GAPDH

Fig.3D

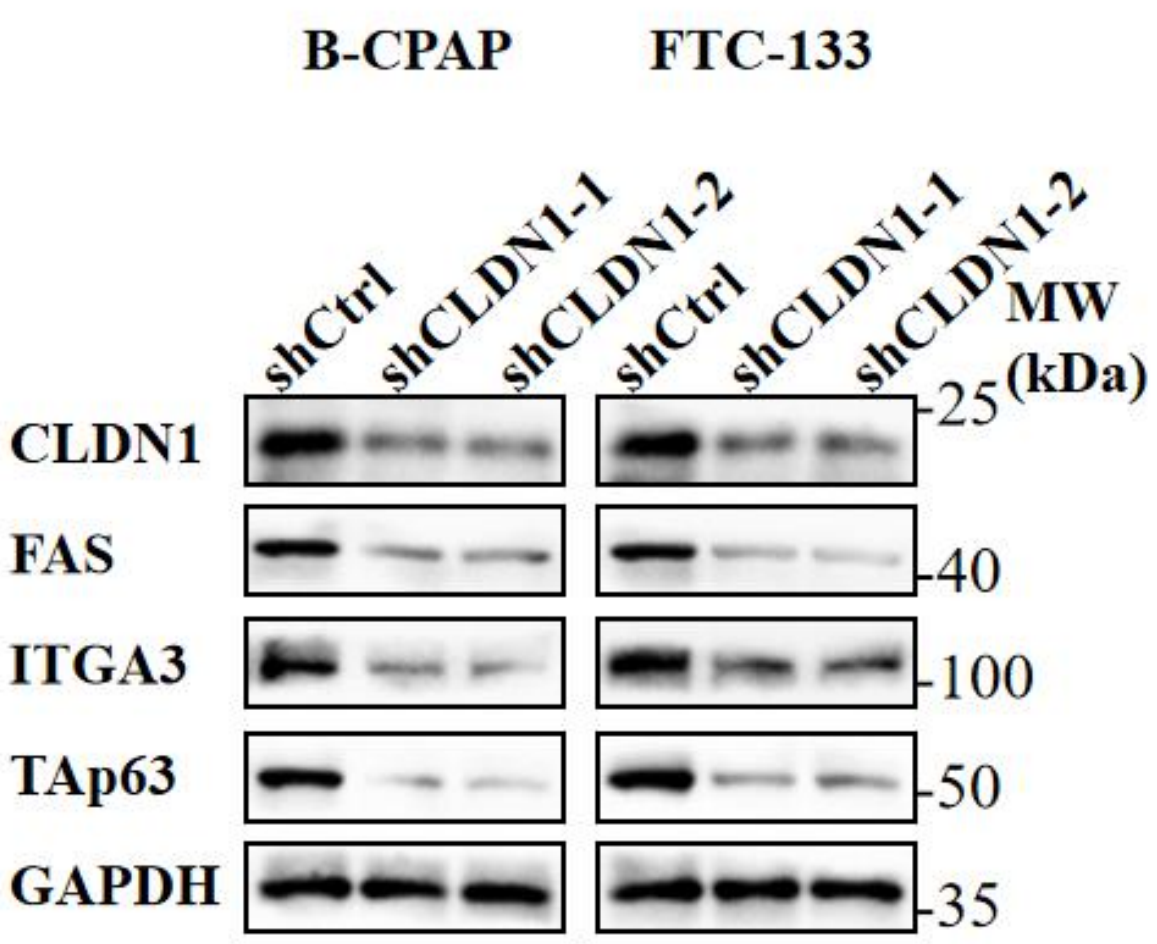

B-CPAP
FTC-133
shCtrl
shCLDN1-1
shCLDN1-2
shCtrl
shCLDN1-1
shCLDN1-2
MW (kDa)
CLDN1
FAS
ITGA3
TAp63
GAPDH
25
40
100
50
35

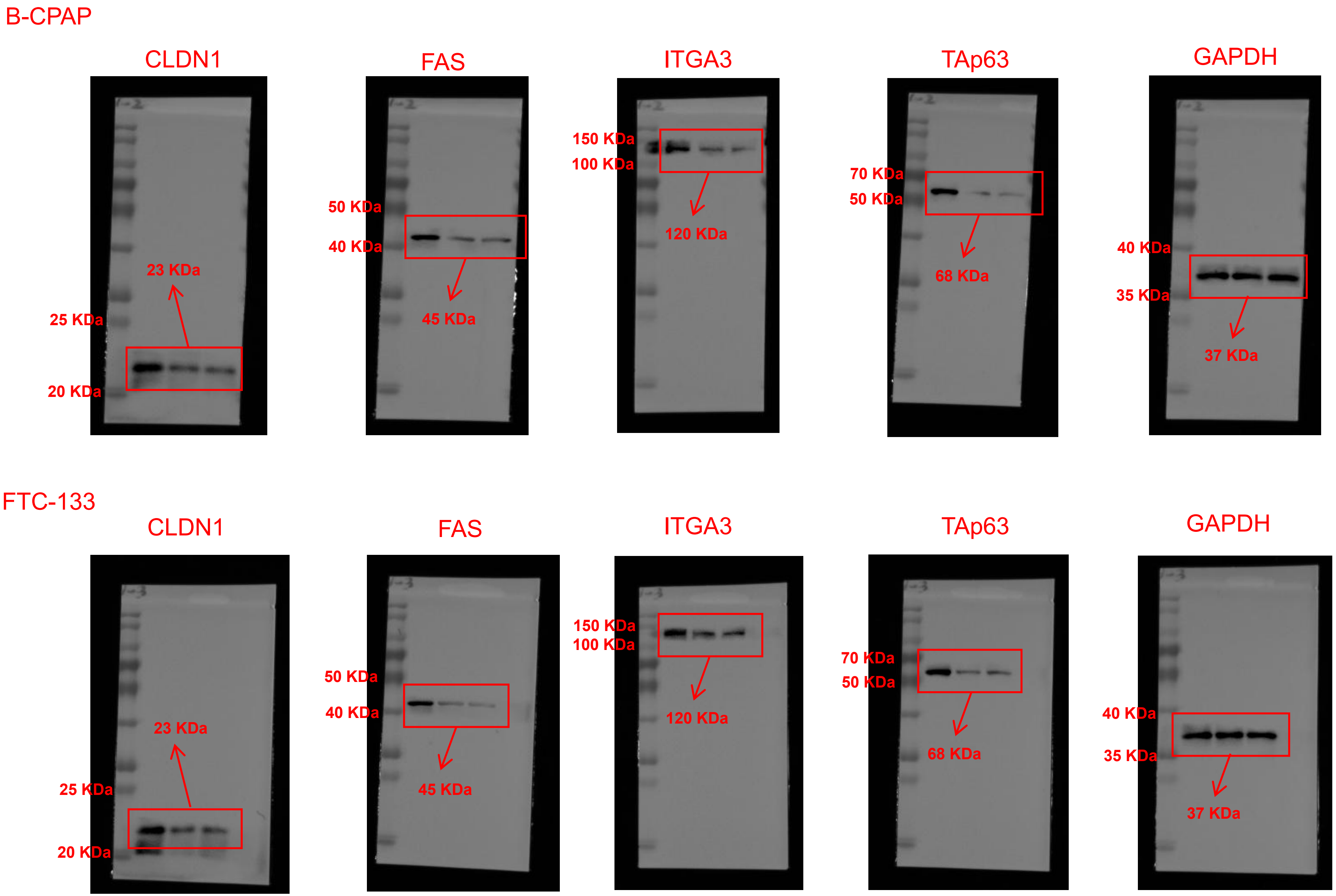

B-CPAP
CLDN1
FAS
ITGA3
TAp63
GAPDH
23 KDa
25 KDa
20 KDa
50 KDa
40 KDa
45 KDa
150 KDa
100 KDa
120 KDa
70 KDa
50 KDa
68 KDa
40 KDa
35 KDa
37 KDa
FTC-133
CLDN1
FAS
ITGA3
TAp63
GAPDH
23 KDa
25 KDa
20 KDa
50 KDa
40 KDa
45 KDa
150 KDa
100 KDa
120 KDa
70 KDa
50 KDa
68 KDa
40 KDa
35 KDa
37 KDa

Fig.3D

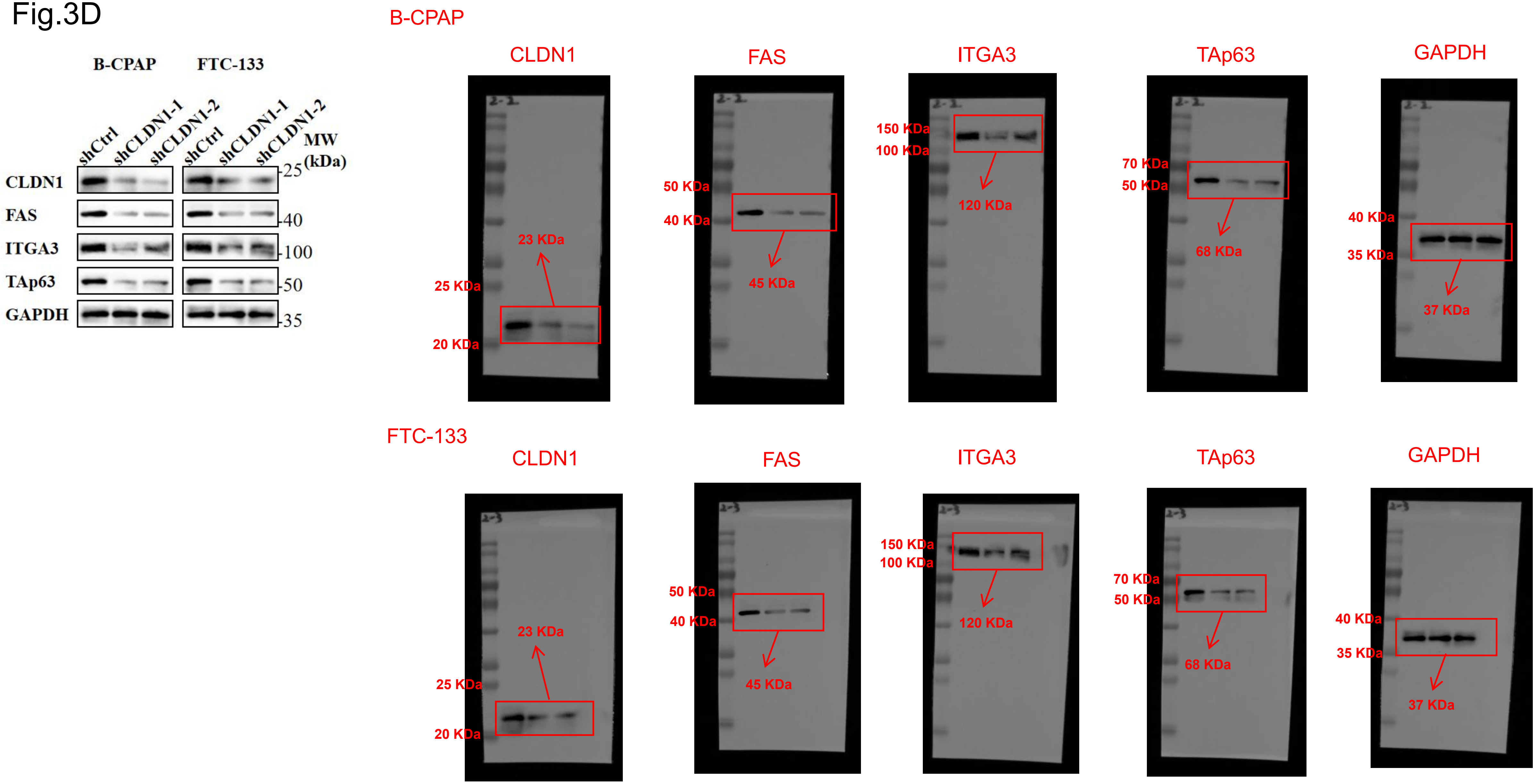
B-CPAP
FTC-133
shCtrl
shCLDN1-1
shCLDN1-2
MW (kDa)
CLDN1
FAS
ITGA3
TAp63
GAPDH
25
40
100
50
35
B-CPAP
CLDN1
FAS
ITGA3
TAp63
GAPDH
23 KDa
25 KDa
20 KDa
50 KDa
40 KDa
45 KDa
150 KDa
100 KDa
120 KDa
70 KDa
50 KDa
68 KDa
40 KDa
35 KDa
37 KDa
FTC-133
CLDN1
FAS
ITGA3
TAp63
GAPDH
23 KDa
25 KDa
20 KDa
50 KDa
40 KDa
45 KDa
150 KDa
100 KDa
120 KDa
70 KDa
50 KDa
68 KDa
40 KDa
35 KDa
37 KDa

Fig.3D

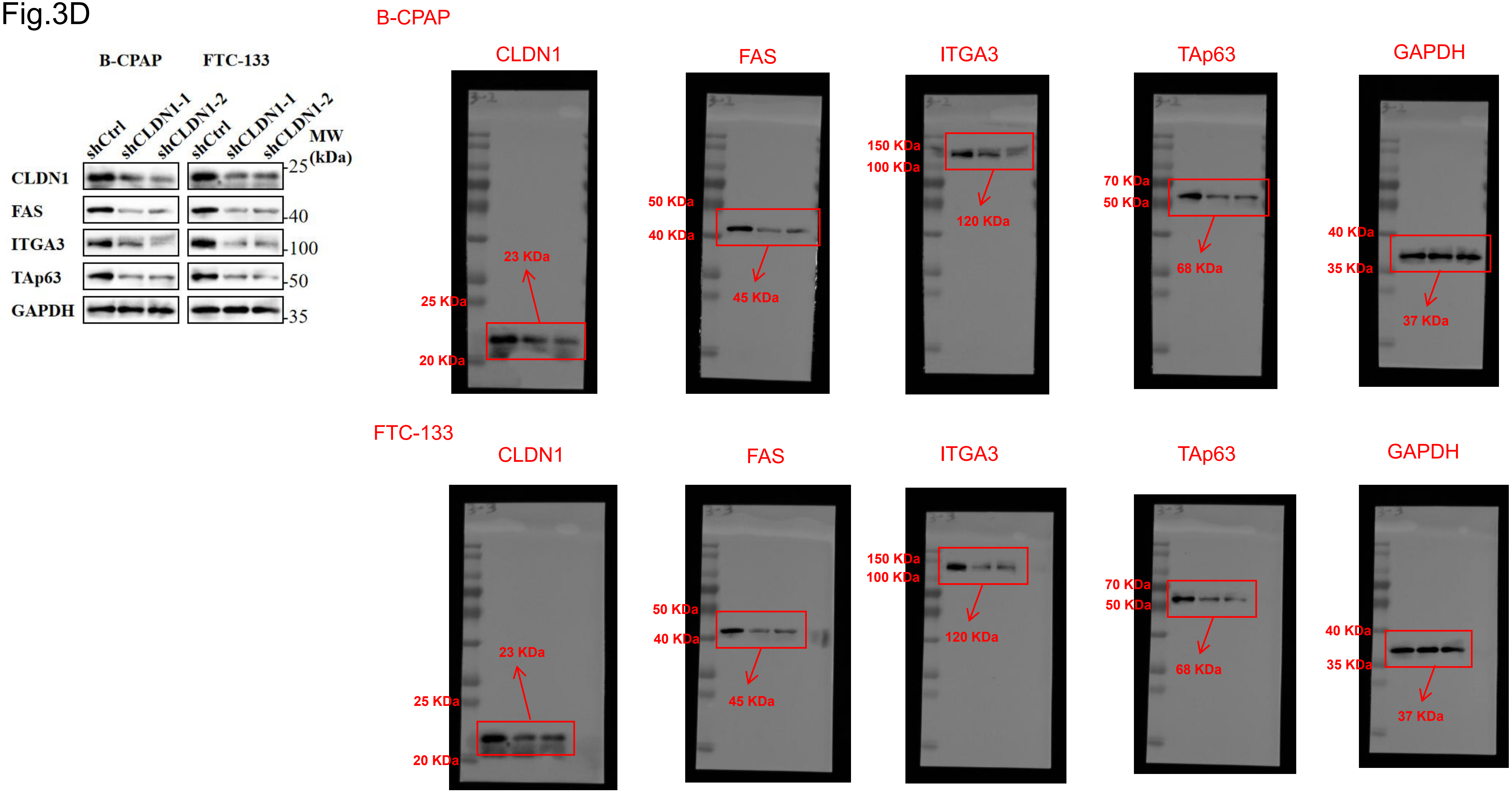

B-CPAP
FTC-133
shCtrl
shCLDN1-1
shCLDN1-2
shCtrl
shCLDN1-1
shCLDN1-2
MW
(kDa)
CLDN1
FAS
ITGA3
TAp63
GAPDH
25
40
100
50
35
B-CPAP
CLDN1
23 KDa
25 KDa
20 KDa
FAS
50 KDa
40 KDa
45 KDa
ITGA3
150 KDa
100 KDa
120 KDa
TAp63
70 KDa
50 KDa
68 KDa
GAPDH
40 KDa
35 KDa
37 KDa
FTC-133
CLDN1
23 KDa
25 KDa
20 KDa
FAS
50 KDa
40 KDa
45 KDa
ITGA3
150 KDa
100 KDa
120 KDa
TAp63
70 KDa
50 KDa
68 KDa
GAPDH
40 KDa
35 KDa
37 KDa

Fig.3E

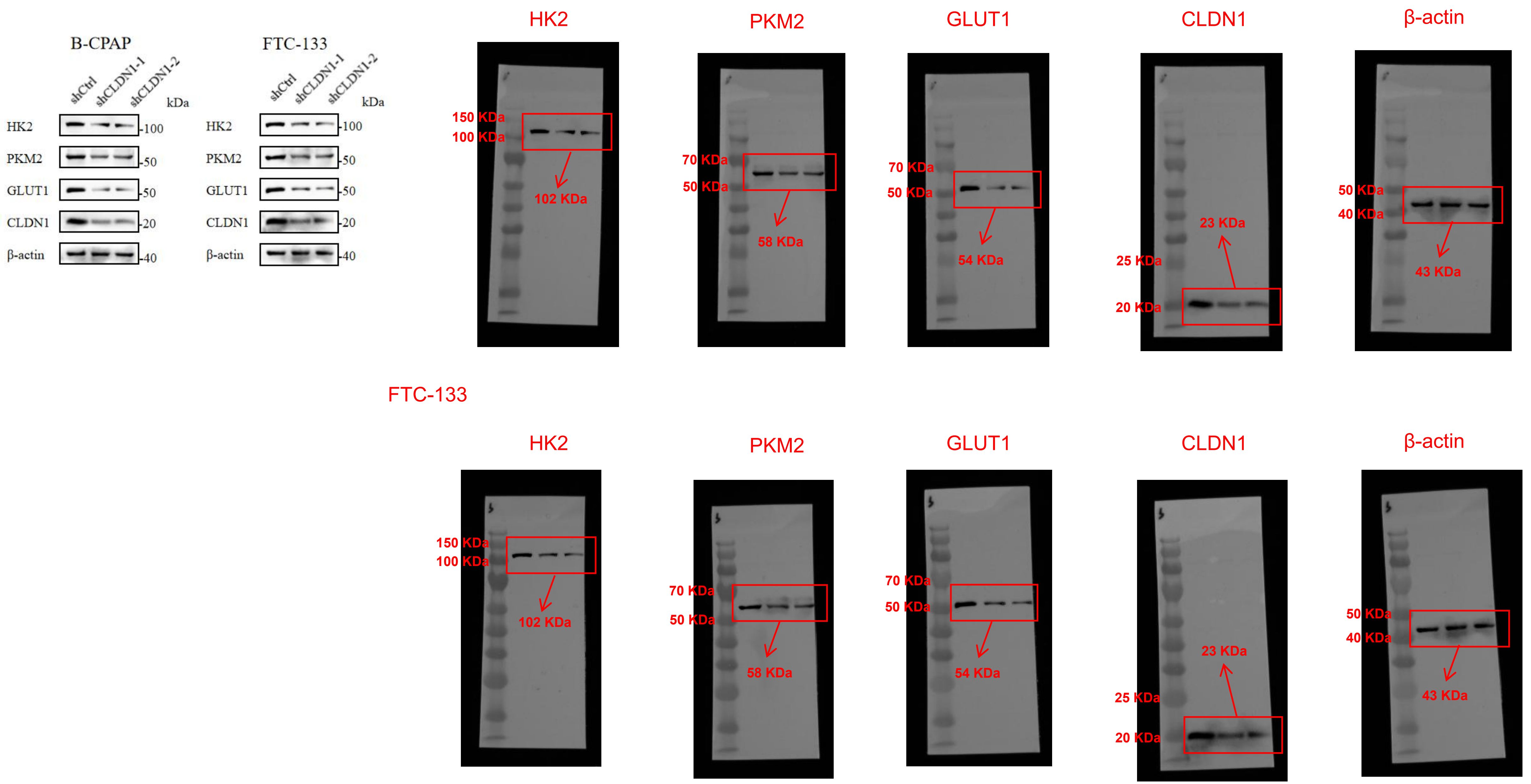

B-CPAP
FTC-133
shCtrl
shCLDN1-1
shCLDN1-2
kDa
HK2
PKM2
GLUT1
CLDN1
β-actin
100
50
20
40
150 KDa
100 KDa
102 KDa
70 KDa
50 KDa
58 KDa
54 KDa
25 KDa
20 KDa
23 KDa
40 KDa
43 KDa

Fig.3E

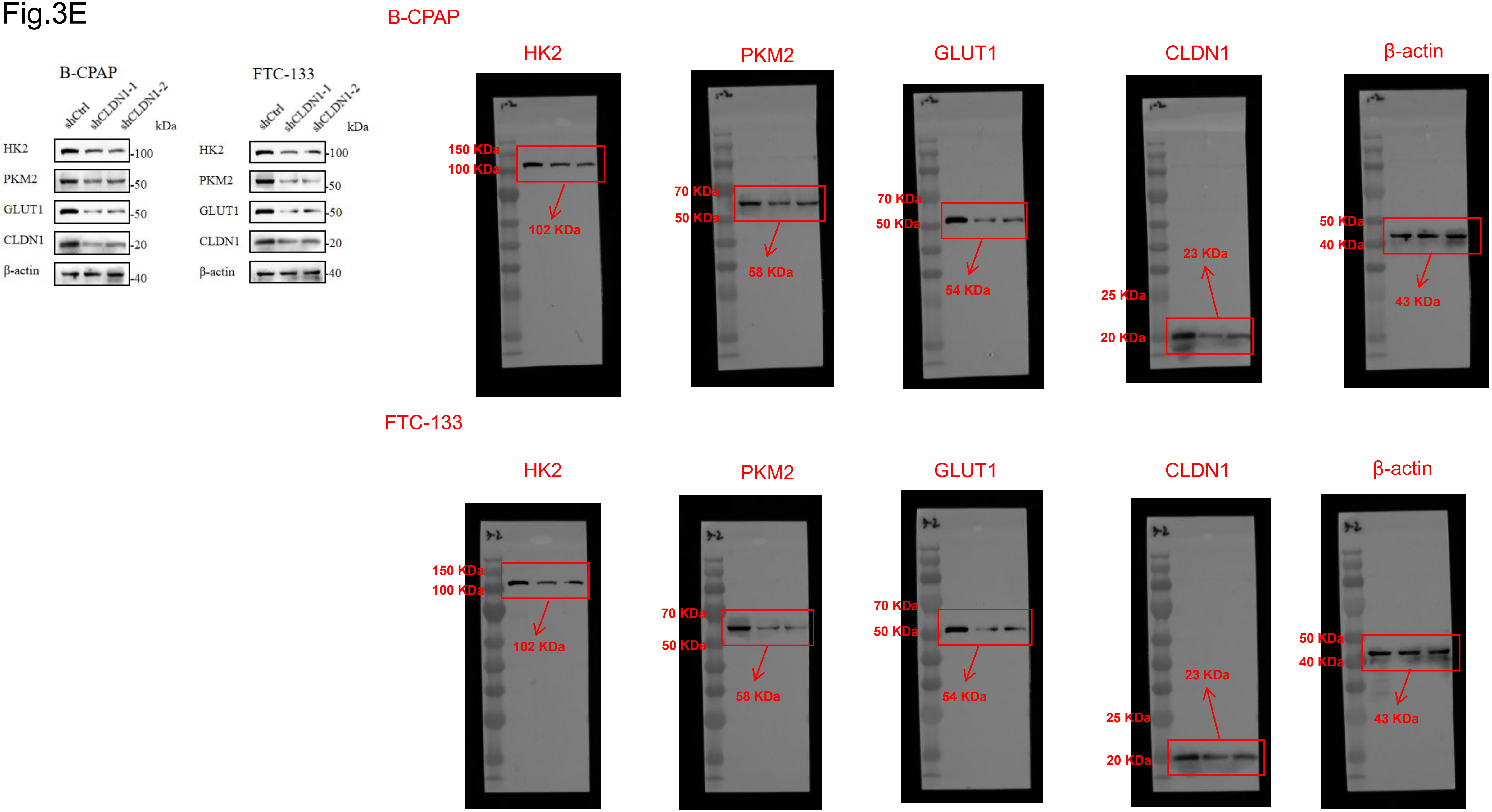

B-CPAP
shCtrl
shCLDN1-1
shCLDN1-2
kDa
HK2
PKM2
GLUT1
CLDN1
β-actin
100
50
50
20
40
FTC-133
shCtrl
shCLDN1-1
shCLDN1-2
kDa
HK2
PKM2
GLUT1
CLDN1
β-actin
100
50
50
20
40
B-CPAP
HK2
150 KDa
100 KDa
102 KDa
PKM2
70 KDa
50 KDa
58 KDa
GLUT1
70 KDa
50 KDa
54 KDa
CLDN1
23 KDa
25 KDa
20 KDa
β-actin
50 KDa
40 KDa
43 KDa
FTC-133
HK2
150 KDa
100 KDa
102 KDa
PKM2
70 KDa
50 KDa
58 KDa
GLUT1
70 KDa
50 KDa
54 KDa
CLDN1
23 KDa
25 KDa
20 KDa
β-actin
50 KDa
40 KDa
43 KDa

Fig.3E

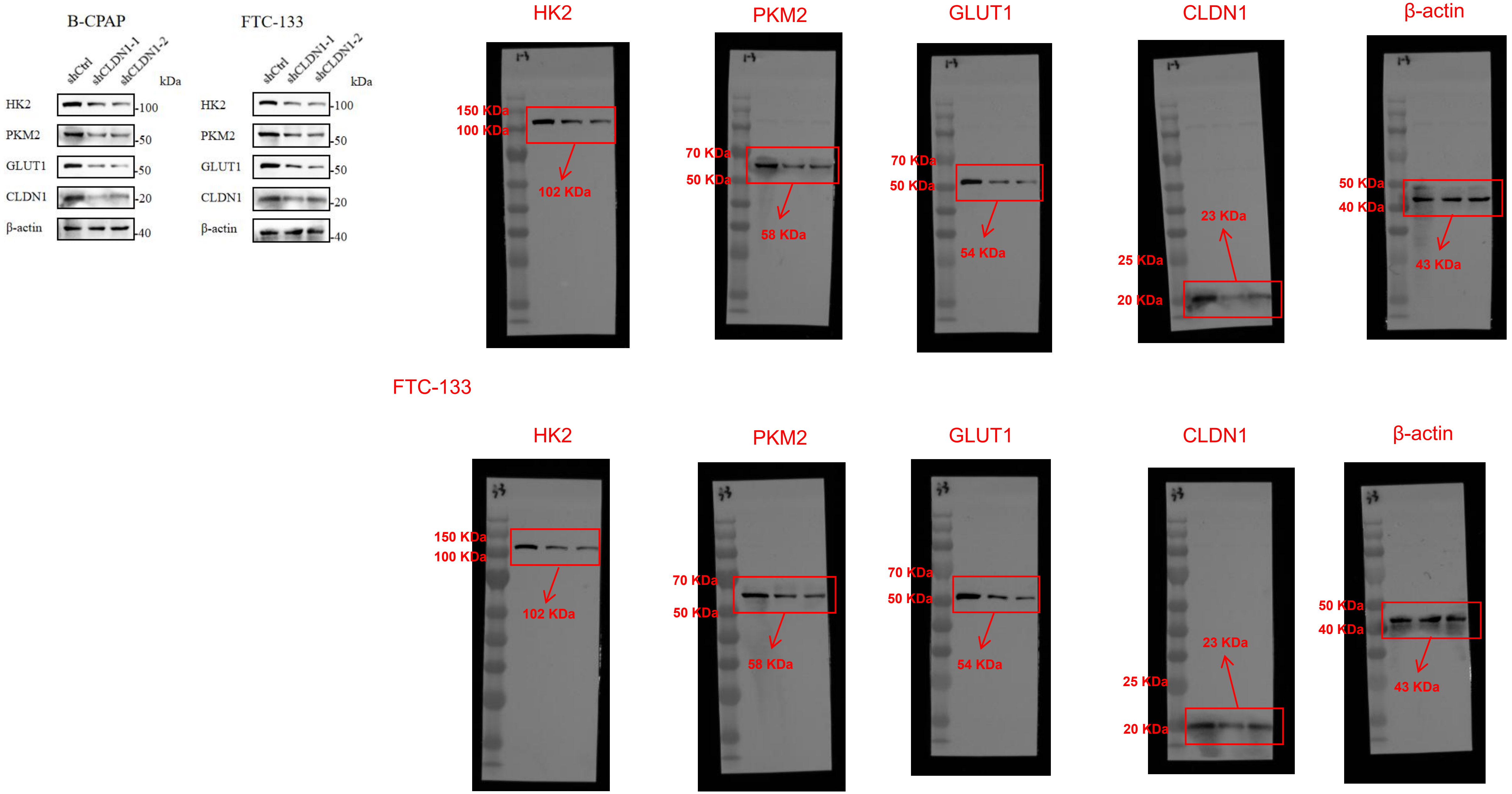

B-CPAP
FTC-133
shCtrl
shCLDN1-1
shCLDN1-2
kDa
HK2
PKM2
GLUT1
CLDN1
β-actin
100
50
50
20
40
150 KDa
100 KDa
102 KDa
70 KDa
50 KDa
58 KDa
54 KDa
25 KDa
20 KDa
23 KDa
40 KDa
43 KDa

Fig.4A

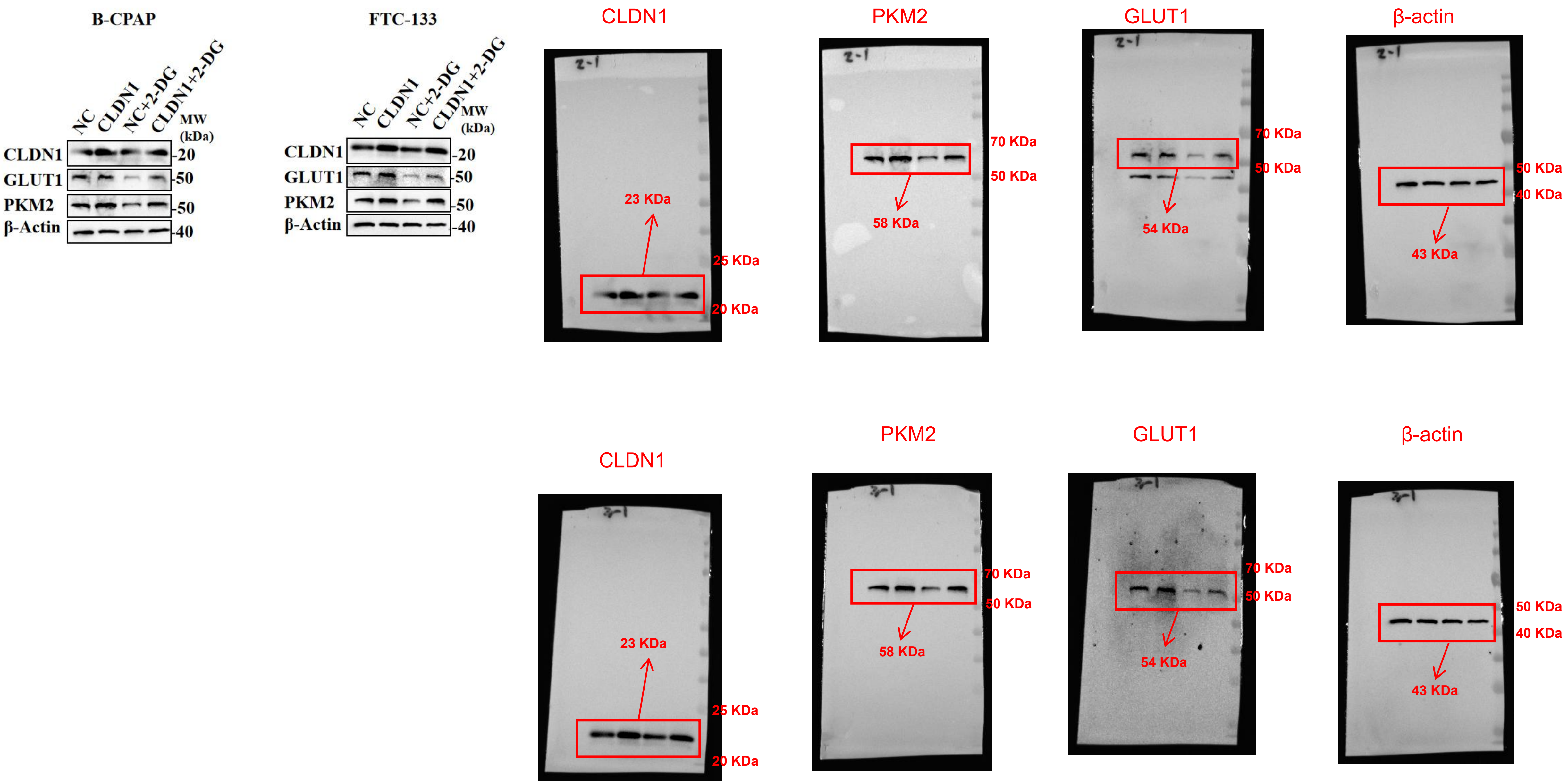
B-CPAP
NC
CLDN1
NC+2-DG
CLDN1+2-DG
MW
(kDa)
CLDN1
GLUT1
PKM2
β-Actin
-20
-50
-50
-40
FTC-133
NC
CLDN1
NC+2-DG
CLDN1+2-DG
MW
(kDa)
CLDN1
GLUT1
PKM2
β-Actin
-20
-50
-50
-40
CLDN1
23 KDa
25 KDa
20 KDa
PKM2
70 KDa
50 KDa
58 KDa
GLUT1
70 KDa
50 KDa
54 KDa
β-actin
50 KDa
40 KDa
43 KDa
CLDN1
23 KDa
25 KDa
20 KDa
PKM2
70 KDa
50 KDa
58 KDa
GLUT1
70 KDa
50 KDa
54 KDa
β-actin
50 KDa
40 KDa
43 KDa

Fig.4A

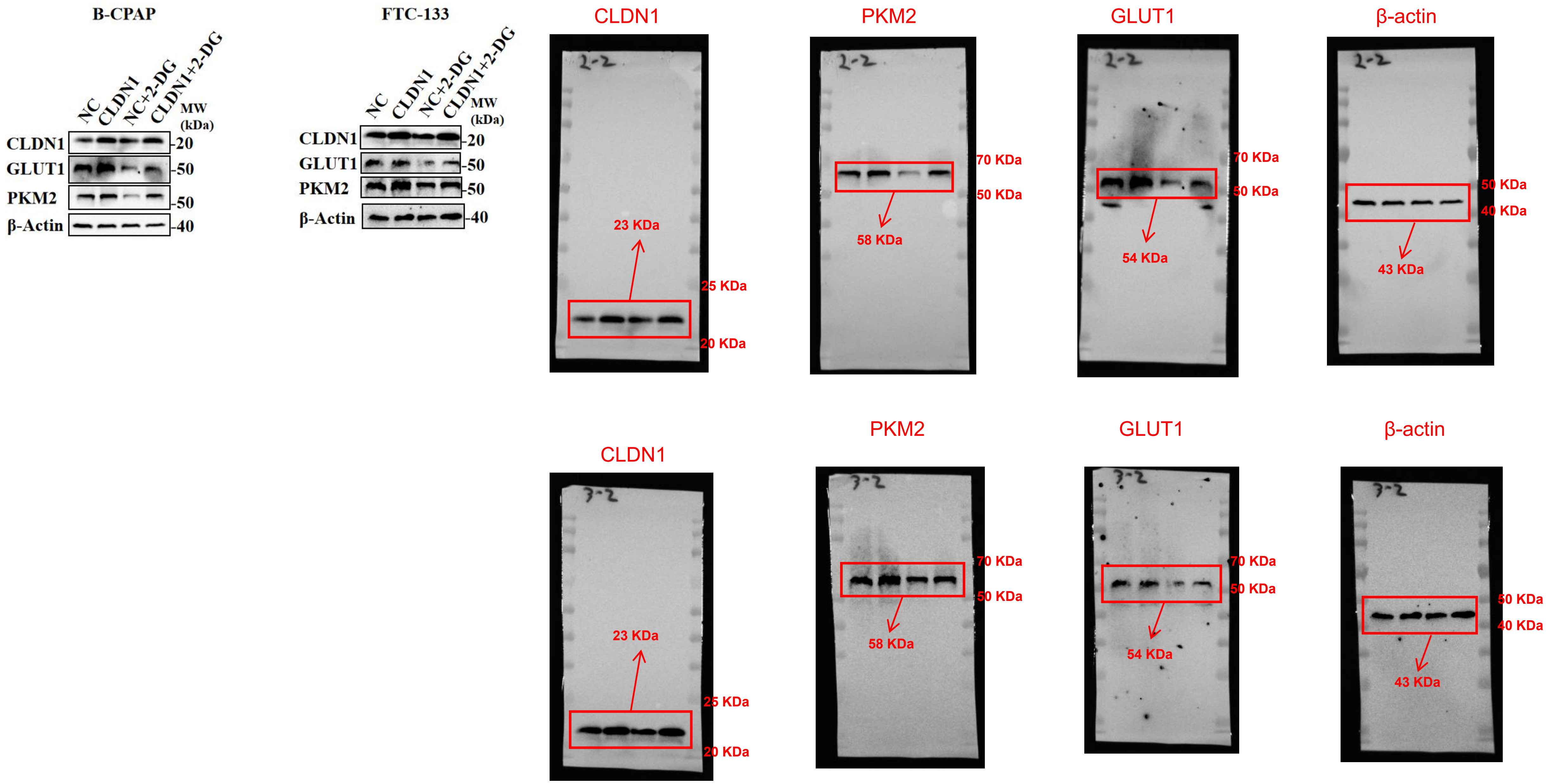

B-CPAP
NC
CLDN1
NC+2-DG
CLDN1+2-DG
MW (kDa)
CLDN1
GLUT1
PKM2
β-Actin
20
50
50
40
FTC-133
NC
CLDN1
NC+2-DG
CLDN1+2-DG
MW (kDa)
CLDN1
GLUT1
PKM2
β-Actin
20
50
50
40
CLDN1
23 KDa
25 KDa
20 KDa
PKM2
70 KDa
50 KDa
58 KDa
GLUT1
70 KDa
50 KDa
54 KDa
β-actin
50 KDa
40 KDa
43 KDa
CLDN1
23 KDa
25 KDa
20 KDa
PKM2
70 KDa
50 KDa
58 KDa
GLUT1
70 KDa
50 KDa
54 KDa
β-actin
50 KDa
40 KDa
43 KDa

Fig.4A

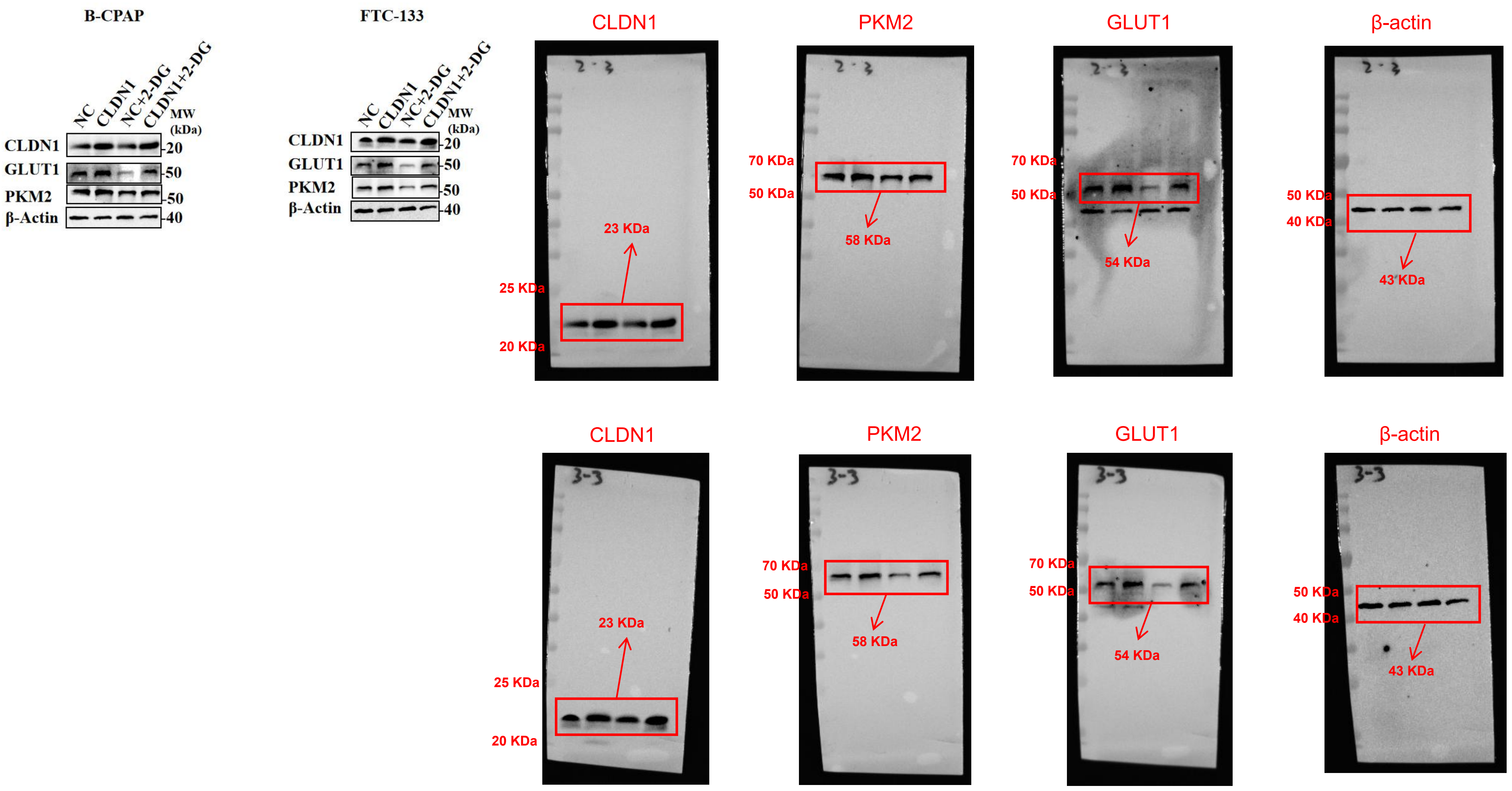
B-CPAP
FTC-133
NC
CLDN1
NC+2-DG
CLDN1+2-DG
MW (kDa)
CLDN1
GLUT1
PKM2
β-Actin
20
50
50
40
CLDN1
PKM2
GLUT1
β-actin
23 KDa
25 KDa
20 KDa
70 KDa
50 KDa
58 KDa
54 KDa
40 KDa
43 KDa

Fig.5F

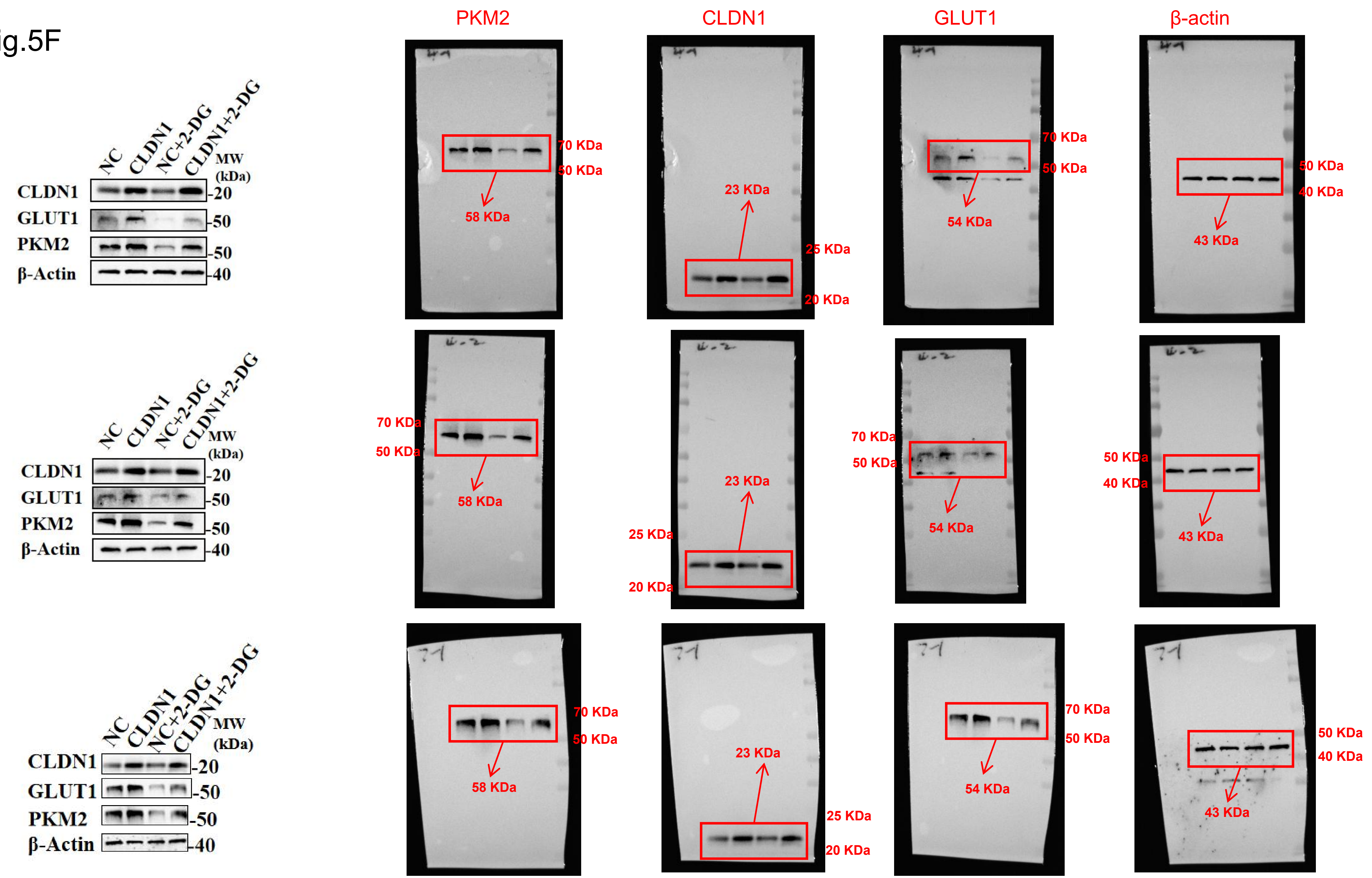

PKM2
CLDN1
GLUT1
β-actin
NC
CLDN1
NC+2-DG
CLDN1+2-DG
MW (kDa)
CLDN1 -20
GLUT1 -50
PKM2 -50
β-Actin -40
70 KDa
50 KDa
58 KDa
23 KDa
25 KDa
20 KDa
54 KDa
40 KDa
43 KDa
